# Massive abiotic methane production in eclogite during cold subduction

**DOI:** 10.1093/nsr/nwac207

**Published:** 2022-09-30

**Authors:** Lijuan Zhang, Lifei Zhang, Ming Tang, Xiao Wang, Renbiao Tao, Cheng Xu, Thomas Bader

**Affiliations:** Ministry of Education Key Laboratory of Orogenic Belts and Crustal Evolution, School of Earth and Space Sciences, Peking University, Beijing 100871, China; Ministry of Education Key Laboratory of Orogenic Belts and Crustal Evolution, School of Earth and Space Sciences, Peking University, Beijing 100871, China; Ministry of Education Key Laboratory of Orogenic Belts and Crustal Evolution, School of Earth and Space Sciences, Peking University, Beijing 100871, China; Ministry of Education Key Laboratory of Orogenic Belts and Crustal Evolution, School of Earth and Space Sciences, Peking University, Beijing 100871, China; Ministry of Education Key Laboratory of Orogenic Belts and Crustal Evolution, School of Earth and Space Sciences, Peking University, Beijing 100871, China; Ministry of Education Key Laboratory of Orogenic Belts and Crustal Evolution, School of Earth and Space Sciences, Peking University, Beijing 100871, China; Ministry of Education Key Laboratory of Orogenic Belts and Crustal Evolution, School of Earth and Space Sciences, Peking University, Beijing 100871, China

**Keywords:** abiotic CH_4_, fluid inclusion, prograde HP-UHP metamorphism, CO_2_, Western Tianshan

## Abstract

Methane (CH_4_) is a critical but overlooked component in the study of the deep carbon cycle. Abiotic CH_4_ produced by serpentinization of ultramafic rocks has received extensive attention, but its formation and flux in mafic rocks during subduction remain poorly understood. Here, we report massive CH_4_-rich fluid inclusions in well-zoned garnet from eclogites in Western Tianshan, China. Petrological characteristics and carbon–hydrogen isotopic compositions confirm the abiotic origin of this CH_4_. Reconstructed P–T–*f*O_2_–fluid trajectories and Deep Earth Water modeling imply that massive abiotic CH_4_ was generated during cold subduction at depths of 50–120 km, whereas CO_2_ was produced during exhumation. The massive production of abiotic CH_4_ in eclogites may result from multiple mechanisms during prograde high pressure-ultrahigh pressure metamorphism. Our flux calculation proposes that abiotic CH_4_ that has been formed in HP-UHP eclogites in cold subduction zones may represent one of the largest, yet overlooked, sources of abiotic CH_4_ on Earth.

## Teaser

Massive abiotic methane can be produced during prograde HP-UHP metamorphism in cold subduction zone, which may represent one of the largest sources of abiotic CH_4_ on Earth.

## INTRODUCTION

Methane (CH_4_) can form on Earth via biotic or abiotic processes. The existence of abiotic CH_4_ has important implications for deep subsurface microbial life, natural gas exploration, global warming and the long-term habitability of Earth [1,2]. Abiotic CH_4_ widely exists in Precambrian crystalline shields, volcanic and geothermal systems, inclusions in crystalline intrusions and serpentinized ultramafic rocks in submarine peridotite-hosted hydrothermal systems, continental ophiolites and peridotite massifs [1–6]. Both experiments [7–9] and theoretical calculations [10,11] indicate that abiotic CH_4_ can form and exist under pressure–temperature (P–T) conditions corresponding to the Earth's upper mantle. Recent experiments have shown that abiotic CH_4_ can be produced in the presence of aqueous fluids at 1.5–3.5 GPa and 300–700°C, P–T conditions that may occur in cold subduction zones [12–14]. Indeed, abiotic CH_4_ has been discovered in fluid inclusions in metasomatized ultramafic ophicarbonates from the cold subduction zones in the Alps [15,16] and Western Tianshan [17], where it formed during serpentinization at forearc depths. However, the formation and pathways of abiotic CH_4_ produced in mafic rocks, especially in eclogites, in natural subduction zones are still unknown.

Carbon is transported from Earth's surface to the mantle in subduction zones [18]. Some of this recycled carbon may be released as CH_4_ and CO_2_ via metamorphism or volcanism. It is controversial whether the vast majority of oceanic carbon is released at forearc to subarc depths [19–22] or deeply subducted [23]. In addition, the amounts of CH_4_ stored in the subducting slab, introduced into the overriding mantle wedge and emitted to the atmosphere through arc magmatism, are poorly constrained. Thus, lacking understanding of the geological, physical and chemical conditions of deep abiotic CH_4_ formation prevents the quantification of these problems.

The Western Tianshan ultrahigh pressure (UHP) metamorphic belt in China, the world's largest cold oceanic subduction zone [24], provides one such case for unraveling the fate of abiotic CH_4_ formed in eclogite at forearc to subarc depths during cold subduction. Coesite relics in various lithologies confirm the estimates of peak metamorphic conditions of 2.7–3.2 GPa and ∼550°C, corresponding to subduction depths of 90–110 km [24]. These rocks represent the most deeply subducted remnant of the Paleo-Asian oceanic lithosphere during the Carboniferous, which is currently exposed along the southern part of the Central Asian Orogenic Belt [24]. Previous petrological and experimental studies showed that CH_4_-bearing fluid inclusions in omphacite from Western Tianshan high pressure (HP) eclogites were formed by the reduction of Fe^2+^-bearing carbonates under low oxygen fugacity [25]. Herein, we first report massive CH_4_-rich primary fluid inclusions in well-zoned prograde garnet from UHP eclogites from the Western Tianshan metamorphic belt, which allow us to determine the P–T–*f*O_2_ conditions of abiotic CH_4_ formation and to estimate the CH_4_ flux released from eclogites during subduction.

## RESULTS

### Prograde metamorphic garnets

**Figure 1.**
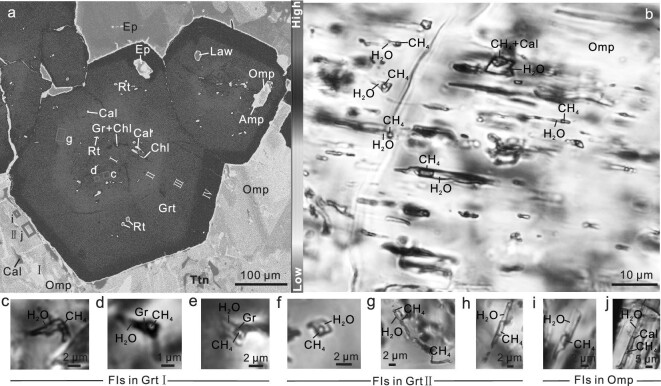
Examples of CH_4_-rich fluid inclusions in eclogite from the Western Tianshan subduction zone. (a) An Fe X-ray map showing the four well-preserved growth zones of garnet (Grt I–IV), abundant CH_4_-rich fluid inclusions preserved in Grt I and Grt II, and the paucity of fluid inclusions in Grt III and Grt IV. (b) Abundant tubular two- or three-phase CH_4_-rich fluid inclusions parallel with the long axis of omphacite (Omp). (c–j) Examples of two- or three-phase CH_4_-rich fluid inclusions in (c–e) Grt I, (f–h) Grt II and (i–j) Omp; gas phases are CH_4_ with minor N_2_, liquid phase is H_2_O, and solid phases (if present) are calcite (Cal) and graphite (Gr). Note that the representative locations of the CH_4_-rich fluid inclusions are marked in numbered squares in (a). Supplementary Figs S2–S6 provide additional element maps, microphotos and locations of CH_4_-rich fluid inclusions.

A CH_4_-rich carbonated eclogite sample (HB142-8) that retains prograde metamorphic mineral assemblages was chosen from >30 samples collected from Western Tianshan (Supplementary Fig. S1). The garnets are Fe-rich, have an almandine molar fraction (*X*_alm_ = Fe^2+^/[Ca + Mg + Fe^2+^ + Mn]) of 52%–73%, and display complex chemical zoning (Fig. 1a; Supplementary Fig. S2 and Table S1). Most garnets show four growth zones: a core region (Grt I) with a high grossular molar fraction (*X*_grs_ = Ca/[Ca + Mg + Fe^2^ + Mn]) of 15%–22%; an inner mantle (Grt II) that has the lowest *X*_grs_ of 11%–15%; an outer mantle (Grt III) where *X*_grs_ increases to 22%–26%; and a rim (Grt IV), which has the highest *X*_grs_ of 29%–31%. The chemical zoning of *X*_alm_ is opposite to that of *X*_grs_. The pyrope molar fraction (*X*_py_ = Mg/[Ca + Mg + Fe^2+^ + Mn]) only increases slightly from Grt I to Grt IV, while the spessartine molar fraction (*X*_sps_ = Mn/[Ca + Mg + Fe^2+^ + Mn]) decreases. This chemical variation in garnet reveals prograde metamorphism during subduction. Single and multiphase solid inclusions such as graphite, Mg-calcite, omphacite, chlorite, lawsonite and rutile commonly occur in Grt I and Grt II. In Grt III and Grt IV, only a few rutile, omphacite, lawsonite and its pseudomorph are present (Fig. 1a; Supplementary Figs S2 and S3). Omphacite also shows zonation from core to rim, with FeO contents decreasing gradually from 7.77 to 2.85 wt% (Fig. 1a; Supplementary Table S1).

### CH_4_-rich fluid inclusions

Abundant CH_4_-rich fluid inclusions are well preserved in Grt I, Grt II and in cores and mantles of omphacite (Omp), but are not present in Grt III, Grt IV and Omp rims (Fig. 1; Supplementary Figs S4–S6). Fluid inclusions in Grt I are mostly elliptic, equiaxial or short columnar (Fig. 1c–e), whereas those in Grt II are mostly tubular, prismatic, dendritic or irregular along with the zoning, and commonly larger than those in Grt I (Fig. 1f–h; Supplementary Fig. S4a–d). Fluid inclusions in Omp occur as isolated parallel tubes or as intragranular clusters, with their long dimensions parallel to the c-axes of the host Omp (Fig. 1b, i and j; Supplementary Fig. S4e and f). These features unequivocally demonstrate the primary nature of the fluid inclusions. These fluid inclusions are different from the secondary fluid inclusions, which were enclosed by olivine during dissolution–precipitation and whose CH_4_ formed due to rarely internal post-entrapment serpentinization [6,26]. Through extensive investigations of samples taken across the Western Tianshan HP-UHP metamorphic belt, we observed that CH_4_-rich fluid inclusions are commonly located in the core and mantle of garnet and omphacite in eclogite, although their prograde zonings are not preserved as well as in the sample HB142-8.

**Figure 2.**
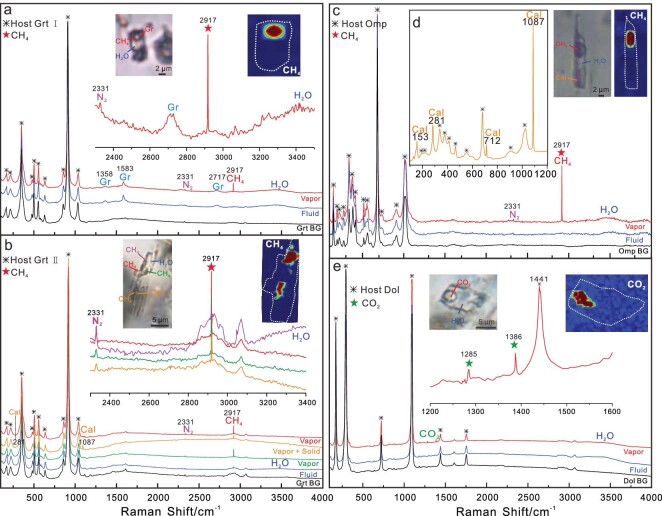
Examples of Raman spectra and hyperspectral confocal Raman maps of CH_4_-rich fluid inclusions. (a–c) CH_4_-rich fluid inclusions in Grt I, Grt II and Omp, respectively. (d) Calcite daughter crystal in a fluid inclusion in Omp. (e) CO_2_-rich fluid inclusion in retrograde ankeritic dolomite. ‘Grt/Omp/Dol BG’ mark the background peaks of the host garnet, omphacite or dolomite, respectively. The inset images are photomicrographs and their corresponding hyperspectral confocal Raman maps of the fluid inclusions, showing the existence of CH_4_ and CO_2._ White dashed lines outline the inclusions.

The CH_4_-rich fluid inclusions in garnet (Grt I and Grt II) and omphacite contain liquid + vapor ± solid daughter crystals at room temperature. The solids are generally very tiny with irregular shapes. The ubiquitous CH_4_ vapor has sharp Raman peaks ranging from 2916 to 2918 cm^−1^, centered mainly at 2917 cm^−1^, and ubiquitous N_2_ marked by weak bands from 2331 to 2328 cm^−1^ (Supplementary Note 1). The fluid phase has the characteristic broad Raman peak for H_2_O at 3440 cm^−1^ (Fig. 2a–c; Supplementary Figs S5 and S6). Daughter crystals are mostly calcite and graphite, and less commonly rutile. The larger solid with a polygonal–prismatic shape has high birefringence and is identified as Mg-calcite with Raman peaks at 1087, 712 and 281 cm^−1^ (Fig. 2d). Most Mg-calcites are very tiny, and have only weak peaks at 1087 cm^−1^. The strong Raman peaks centered at 1583 and 1356 cm^−1^ indicate the presence of well-crystallized graphite (Fig. 2a; Supplementary Figs S5 and S6). Abundant CO_2_-rich fluid inclusions, which are mostly larger than the CH_4_-rich fluid inclusions, are present in retrograde ankeritic dolomite (for details see Supplementary Note 1 and Fig. S7). The CO_2_ vapor has two Raman peaks at 1285 and 1386 cm^−1^; the fluid phase also has the broad Raman peak for H_2_O at 3440 cm^−1^ (Fig. 2e; Supplementary Fig. S8). No H_2_ was detected in any of the fluid inclusions.

### Stable C-H isotopic compositions of CH_4_

**Figure 3.**
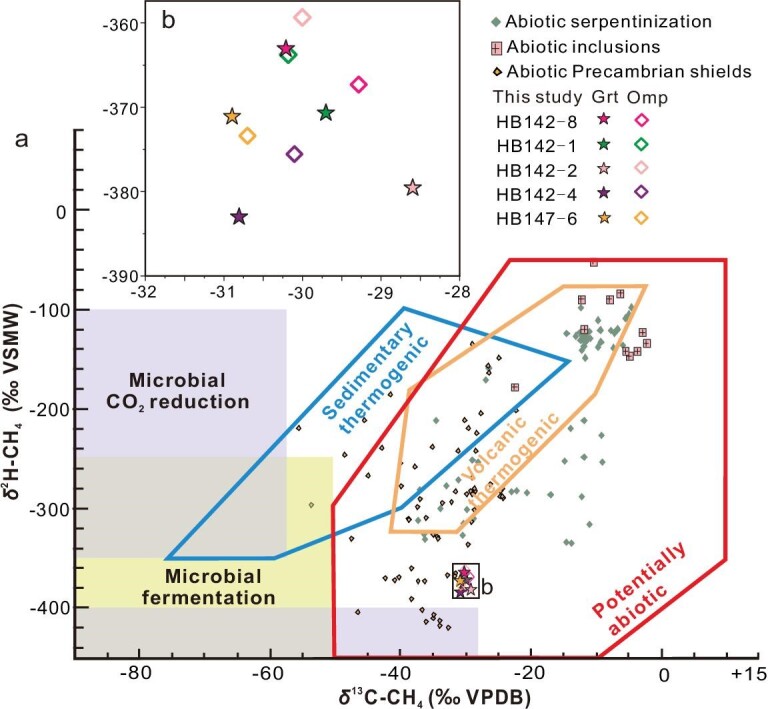
Stable C and H isotope compositions of CH_4_. (a) The global summary of CH_4_ with outlines of the microbial, sedimentary thermogenic, volcanic thermogenic and potentially abiotic areas taken from reference [27]. New data for CH_4_ gas preserved in eclogites from Western Tianshan plot in the field of potentially abiotic origin. Abiotic CH_4_ data for serpentinization, inclusions in crystalline intrusions and Precambrian shields are shown for comparison [1]. (b) The enlargement of the rectangle in (a) clearly shows the δ^13^C and δ^2^H values in both garnet and omphacite from the eclogite samples from the Western Tianshan.

The δ^13^C and δ^2^H values of CH_4_ (see Methods) in both garnet and omphacite from the Western Tianshan eclogites are rather homogeneous, relatively ^13^C enriched and ^2^H depleted. The δ^13^C values of CH_4_ in garnet vary from −30.9‰ to −28.6‰ and δ^2^H values span −383.0‰ to −363.1‰. The δ^13^C and δ^2^H values of CH_4_ in omphacite are −30.7‰ to −29.3‰ and −375.6‰ to −359.5‰, respectively (Fig. 3; Supplementary Table S2). Our δ^13^C and δ^2^H data plot entirely in the field of potentially abiotic CH_4_ origin, none overlapping with the microbial, volcanic and sedimentary thermogenic CH_4_ fields (Fig. 3) [27].

### Phase equilibrium modeling and oxygen fugacity calculation

**Figure 4.**
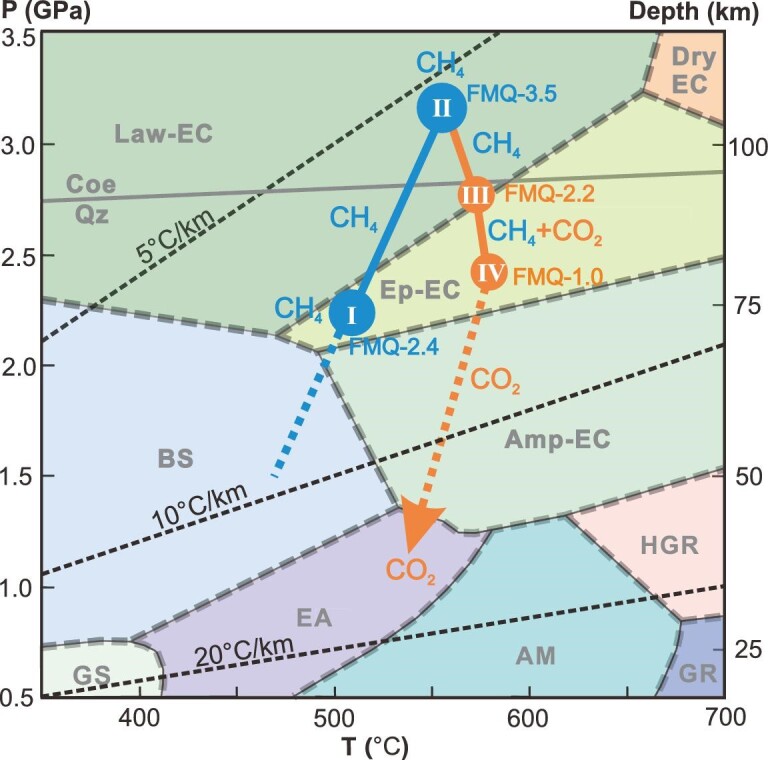
P–T–*f*O_2_-fluid evolution trajectory of the representative CH_4_-rich eclogite HB142-8. Circles with I to IV are P–T conditions corresponding to the four growth zones of garnet obtained by Perple_X in the system of MnNCKFMASCHO (Supplementary Fig. S9). The blue and orange lines with an arrow represent the prograde and retrograde HP-UHP metamorphic path, respectively. The marked oxygen fugacity (*f*O_2_) values were calculated by paired garnet and corresponding omphacite inclusions captured in Grt I–IV (Supplementary Tables S1, S3 and S4).

The growth zoning of garnet from the Western Tianshan eclogite is revealed to record an amazing P–T–*f*O_2_ trajectory (Fig. 4), which we quantify with phase equilibrium modeling and *f*O_2_ calculation (see Methods, Supplementary Fig. S9 and Tables S3–S5). Grt I reflects prograde metamorphism at 2.1–2.3 GPa, 500–530°C (∼70 km), with 2.4 log units below the Fayalite–Magnetite–Quartz buffer (FMQ). Grt II shows UHP peak metamorphism at 3.2–3.4 GPa, 540–560°C (∼110 km), in accord with the ubiquity of coesite in the same unit [24], and the *f*O_2_ decreases to the lowest FMQ value of −3.5. The temperature estimates are consistent with the results of 530–560°C obtained through Raman spectra of graphite coexisting with CH_4_ [28]. Grt III and Grt IV reveal heating with decompression to 2.7–2.8 GPa at 560–580°C, and 2.3–2.5 GPa at 570–590°C, respectively; the *f*O_2_ increases to FMQ of −2.2 and −1.0 for Grt III and Grt IV, respectively.

### Metamorphic aqueous fluid composition by DEW modeling

**Figure 5.**
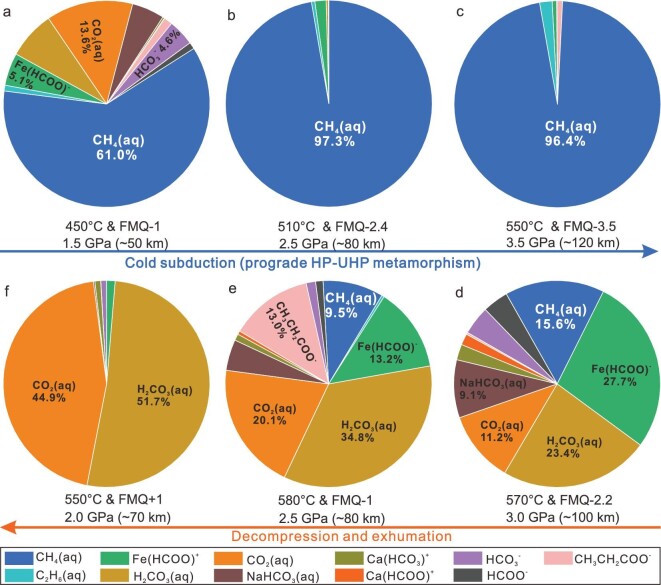
Compositional variation of aqueous carbon species in eclogitic fluid along the P–T–*f*O_2_-fluid evolution trajectory in Fig. 4. (a–c) Carbon species are dominated by reduced aqueous CH_4_ during the cold subduction (prograde HP-UHP metamorphism). (d–f) The proportion of oxidized carbon species gradually increases during the decompression and exhumation.

In order to quantify the compositional evolution of aqueous fluid in the Western Tianshan subduction zone, the DEW (Deep Earth Water) model [29,30] was used (see Methods). The metamorphic fluid compositions largely depend on the P–T–*f*O_2_ conditions (Fig. 5). Carbon species are dominated by reduced aqueous CH_4_, which increased from 61% at ∼50 km (Fig. 5a) to 97% at ∼80 km (Fig. 5b) and ∼120 km (Fig. 5c), along with prograde HP-UHP metamorphism. This is consistent with our observation of massive CH_4_ in prograde Grt I–II and Omp (Fig. 1 and Supplementary Figs S4–S6). The abiotic CH_4_ actually began to form in the blueschist facies at depths of <50 km, which is not recorded by garnet. By contrast, the amount of CH_4_ suddenly decreased during the decompression and exhumation, while the proportion of oxidized carbon species, such as CO_2_ and H_2_CO_3_, gradually increased (Fig. 5d–f). This agrees with the absence of CH_4_ in Grt III and Grt IV, and the presence of CO_2_ in retrograde ankeritic dolomite (Supplementary Figs S7 and S8), although we did not detect other oxidized carbon species, such as H_2_CO_3_ and CH_3_CH_2_COO^−^. Consequently, the calculated fluid composition is basically consistent with our observation.

## DISCUSSION AND IMPLICATIONS

### The variation of carbon species and the redox properties in subduction-zone metamorphic fluids

The combination of the petrological observations (Figs 1 and 2 and Supplementary Figs S1–S8) and thermodynamic models (Figs 4 and 5 and Supplementary Fig. S9) leads to a complete P–T–*f*O_2_-fluid evolution trajectory for the CH_4_-bearing eclogites. During the prograde metamorphism from the blueschist facies at ∼50 km to eclogite facies at ∼70 km (Grt I), the carbon species in the fluids were dominated by reduced CH_4_, and the proportion of CH_4_ increased from ∼61% to ∼97%. From HP eclogite facies at ∼70 km (Grt I) to the UHP eclogite facies at ∼120 km (Grt II), the carbon species in the aqueous fluid was mainly CH_4_, and it remained at the maximum of ∼97%. This is consistent with the behavior of sulfur in the same area [31]: reduced H_2_S and HS^−^ prevailed in the fluids, and their release reached its climax at ∼90 km depth [31]. During the exhumation with heating and decompression (Grt III–Grt IV), the fluid evolved into a CO_2_–CH_4_-bearing aqueous fluid and the oxygen fugacity increased to FMQ-1. This is consistent with the rare presence of fluid inclusions with coexisting CO_2_ and CH_4_ in the core of a retrograde ankeritic dolomite (Supplementary Note 1 and Fig. S8h). With the progression of the decompression and the increase of the oxygen fugacity, the carbon species in the fluids completely changed into CO_2_, and CO_2_ perpetuated through the epidote–amphibole facies to even shallower depths during the exhumation, as recorded by CO_2_-rich fluid inclusions in the rim of the retrograde ankeritic dolomite (Fig. 2e; Supplementary Figs S7 and S8). Therefore, our study provides new clues with regard to the controversy over the reduced [31,32] or oxidized [33–36] state of the subduction zone fluids. Our study demonstrates that the compositions and the redox properties of subduction zone fluids vary with the P–T–*f*O_2_ conditions, and the fluids are neither always oxidized nor perpetually reduced. Therefore, it is necessary to characterize the fluid compositions and properties for each distinct metamorphic stage in subduction zones.

### The mechanisms and origin of CH_4_

The reduction of Fe^2+^-bearing carbonates under low oxygen fugacity is an important mechanism for the formation of abiotic CH_4_ in subduction zones, and experiments reproduced it [25]. Textures imply that such a metamorphic reaction (more precisely, the decomposition of relict prograde ankeritic dolomite to calcite/aragonite, magnetite and graphite) proceeded in the samples studied herein (Supplementary Note 1 and Supplementary Fig. S7a). This substantiates that the reaction ankeritic dolomite + H_2_O => calcite/aragonite + magnetite + graphite + CH_4_ was the main reaction for CH_4_ production in the Western Tianshan eclogites. In addition, the inclusions of graphite, Mg-calcite, lawsonite and chlorite in the prograde Grt I and Grt II (Supplementary Fig. S2 and S3) point to another possible reaction for CH_4_ production in the Western Tianshan eclogites: graphite + lawsonite +/− chlorite = > calcite/Mg-calcite + epidote + CH_4_ + H_2_O. This reaction was verified by experiments, in which the abiotic hydrocarbons were produced by the reduction of graphite or diamond with aqueous fluids at high-P and low-T conditions [12,37–39]. Moreover, carbonate dissolution is considered as the main decarbonization mechanism in subduction zones [19,34,35]. We cannot exclude the fluid-mediated dissolution and precipitation of carbonates because of the common occurrence of calcite in the fluid inclusions (Figs 1b, j, 2b–d and Supplementary Figs S5 and S6). Therefore, besides the reduction of Fe^2+^-bearing carbonates, metamorphic reactions of graphite and carbonate dissolution may also be efficient mechanisms that produced abiotic CH_4_ during the prograde HP-UHP metamorphism in the Western Tianshan eclogites. By contrast, during the exhumation, the increase of oxygen fugacity favors the production of CO_2_: CH_4_ released by Grt I, Grt II and Omp was converted into CO_2_ subsequently, which was captured by retrograde ankeritic dolomite while *f*O_2_ increased (Supplementary Figs S7 and S8).

The petrographic textures and the mechanisms for the formation of CH_4_ outlined above do not involve organic carbon, pointing to an abiotic origin of the CH_4_ in eclogites. Moreover, our δ^13^C and δ^2^H data plot into the field of abiotic origin, but they are different from the abiotic CH_4_ formed in crystalline intrusions, Precambrian shields and serpentinization (Fig. 3) [1,27]. Reversely, if the CH_4_ in our inclusions has a biotic origin, the hydrogen isotope composition of CH_4_ and H_2_O should have re-equilibrated during entrapment. Grt II recorded a temperature of up to 560°C (Fig. 4), and δ^2^H of the coexisting H_2_O is estimated at about −270‰ [40]. Such a depleted hydrogen isotope composition in a subduction zone fluid system is not expected [41]. The same scenario applies to carbon isotopes. Hydrogen and carbon isotope compositions are in disequilibrium with the surrounding hydrogen and carbon sources, indicating the CH_4_ formed by abiotic origin. Therefore, the petrological characteristics and C–H isotopic compositions both confirm the abiotic origin of the CH_4_ in this study.

### Cold subduction boosts abiotic CH_4_ production

Abiotic CH_4_ has increasingly been recognized in HP-UHP metamorphic rocks from subduction zones. CH_4_-rich fluid inclusions occur in high pressure-low temperature (HP-LT) ophicarbonates from the Western Alps [15,16], the Alpine Corsica [16] and the Western Tianshan [17]; in HP/UHP-LT eclogites from the Western Tianshan [25 and this study]; in metaperidotites from the Appalachian [42] and the North Qilian HP-LT metamorphic belt [43]; and in continental-type HP/UHP-MT eclogites (but solely in local reduced environments) [44,45]. From these examples it seems that cold subduction zones with HP/UHP-LT conditions favor the production of abiotic CH_4_.

The thermal structure differs distinctly between cold and warm subduction zones [46]. For example, cold subduction zones are characterized by much deeper serpentinization (∼100 km depth) than warm subduction zones (<35 km) [16]. By contrast, the serpentinization of the mantle wedge is predicted to be more pronounced in warm subduction zones compared to cold ones [16,47]. These conditions favor high H_2_-CH_4_ concentrations in fluid in cold subducting slabs, but high H_2_ and CH_4_ fluxes in the mantle wedge in warm subduction zones [16]. Consequently, cold subducting slabs are more reduced and advantageous for CH_4_ production than warm subducting slabs at depths of 35–100 km; this concurs with the massive CH_4_ production in eclogites from forearc to subarc depth observed herein (Fig. 4). Concordantly, most recent experiments indicate that CH_4_-bearing reduced fluid can be produced at P–T conditions (1.5–3.5 GPa and 300–700°C) comparable to cold subduction zones [12–14]. Experiments and simulation calculations also predicted that the production of abiotic CH_4_ is facilitated at low-temperature conditions (T < 1500 K), whereas dissociation to higher hydrocarbons proceeds at high-temperature conditions (T > 1500 K) [7–11,39]. The DEW model calculation also shows that CH_4_ has a much higher proportion than CO_2_ in aqueous eclogitic fluids in cold subduction zones (T < 700°C) compared to warm subduction zones (T > 700°C) at fixed conditions of 5 GPa and FMQ-2 [48]. Thermodynamic calculations also demonstrated that CH_4_ is stabilized in cold subduction zones relative to CO_2_, especially in graphite-saturated C-O-H systems like the one studied herein [37]. All this evidence indicates that cold subduction zones are more conducive to the formation of abiotic CH_4_ than warm subduction zones. In fact, the *f*O_2_ of the subducted slabs are remarkably heterogeneous due to varying degrees of alteration at the seafloor, and fluid/rock ratios and redox budgets should be considered [36,37,49,50]. Consequently, we conclude that cold subduction zones can boost abiotic CH_4_ production at favorable *f*O_2_ conditions and bulk rock compositions.

### Abiotic methane flux released from HP-UHP eclogites during cold subduction

Our data permit provisional estimates of the abiotic CH_4_ flux released from eclogites in worldwide modern subduction zones. Based on the moles of CH_4_ per kilogram of H_2_O calculated with the DEW model at specific P–T–*f*O_2_ conditions during prograde metamorphism from 50 km to 120 km (Fig. 5a–c; Supplementary Table S6), the amount of H_2_O released from the subducting slab along our prograde P–T–*f*O_2_ trajectory (∼4 wt%) [51] and the total mass of eclogites subducted annually, the calculated CH_4_ flux released from eclogites could be 10.85 Mt/y (Supplementary Note 2 and Table S7). We also considered the amount of H_2_O lost annually from global mafic rocks from depths of 50 km to 100 km (2.865 × 10^14^ g/y) [46]: the multiplication of this value with the average moles of CH_4_ per kilogram of H_2_O (2.353 mole/kg) yields a total released CH_4_ flux of 10.79 Mt/y. The estimated results obtained from the two different methods are nearly the same, which indicates that the results are reliable. The CH_4_ flux released from the mafic eclogites (∼10.8 Mt/y) much exceeds the abiotic CH_4_ production at mid-ocean ridges (1.1–1.9 Mt/y) [52] and by HP serpentinization worldwide (2.3 × 10^−3^ to 1 Mt/y) [16].

**Figure 6.**
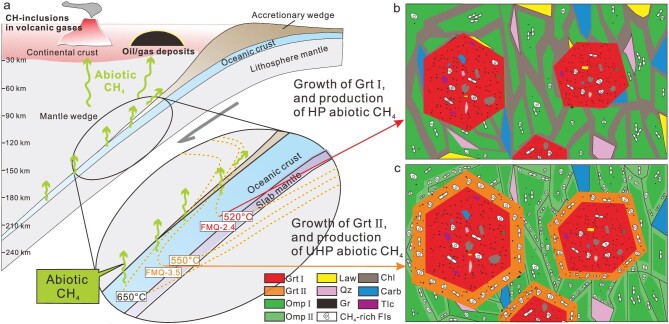
Illustration of the abiotic CH_4_ production during prograde HP-UHP metamorphism in an oceanic subduction zone (a). The subduction zone comprises oceanic crust, slab mantle, mantle layers and continental crust. On the right, the images (b and c) show the entrapment of CH_4_-rich fluid inclusions during the growth of prograde Grt I, Grt II and Omp, corresponding to P–T–*f*O_2_ conditions during deep subduction (enlarged image in the middle). The abiotic CH_4_ produced during the prograde HP-UHP metamorphism migrates upwards to shallow depths, potentially contributing to shallow gas or oil deposits or returning from subarc depths to the surface by degassing from arc volcanoes.

We have discussed that the reduced and cold subduction zones favor the production of abiotic CH_4_ (see above). Therefore, to conservatively estimate abiotic CH_4_ flux released from eclogites during cold subduction, we also considered cold paleo-subduction zones with reduced environments. In the case of the ancient Southern Tianshan cold subduction zone, the CH_4_ flux released from the eclogites during the prograde HP-UHP metamorphism at depths of 50–120 km is 0.49 Mt/y (Supplementary Note 2 and Table S7). The subduction-related production of CH_4_ ± H_2_ has been recorded in ophicarbonates and metaperidotites from the Alps, Western Tianshan, North Qilian and Appalachian at FMQ −6.0 to FMQ −2.0 [15–17,42,43], which is within the range of FMQ-2 to FMQ-4.5 known to permit the stability of CH_4_ [37,48,50]. Eclogites in such reduced and cold subduction zones [15–17,42,43] are capable of producing abiotic CH_4_, and the estimated CH_4_ flux could be 1.76 Mt/y (Supplementary Note 2 and Tabl  S7). The above estimates do not include either abiotic CH_4_ from the HP serpentinization [15–17], or the potential contribution from the metapelitic schists that experienced similar P–T–*f*O_2_ paths as the HP-UHP eclogites [24]. Consequently, the subducted cold oceanic crust may be an important, yet overlooked, source of abiotic CH_4_, which cannot be ignored in estimates of the global carbon flux. The released abiotic CH_4_ might migrate upwards and affect the redox state of the overlying mantle wedge. Potentially, it contributes to natural gas deposits at shallow depths, and/or returns to the atmosphere by degassing through arc volcanoes, further influencing the climate and the environment (Fig. 6).

## METHODS

### Major element compositions and Raman spectroscopy analysis

Major element and Raman spectroscopy analysis were both conducted at Peking University. The former was performed on a JEOL JXA-8230 electron microprobe using a 15 kV acceleration voltage and 10 nA beam current. The beam diameter was set to 2 μm for silicates and 10 μm for carbonates. Raman spectroscopy was performed on a HORIBA Jobin Yvon confocal LabRAM HR Evolution micro-Raman system equipped with a frequency doubled green Nd-YAG laser (532 nm), a 100× short-working distance objective, and a stigmatic 800 mm spectrometer with a 600 groove/mm grating. The laser spot size was focused to 1 μm and the accumulation time varied between 60 and 120 s. The estimated spectral resolution was greater than 1.0 cm^−1^ and the calibration used synthetic silicon. Hyperspectral Raman images were collected along a regular grid of points, nearly equidistant in both directions, with a computer-controlled, automated X–Y mapping stage. All Raman data are presented in the original raw spectra state, without any baseline subtraction correction.

### Carbon and hydrogen isotope analyses

The δ^13^C and δ^2^H values are expressed as δ^13^C (‰) = [(^13^C/^12^C)_sample_/(^13^C/^12^C)_VPDB_ − 1] × 1000, and δ^2^H = [(^2^H/^1^H)_sample_/(^2^H/^1^H)_VSMOW_ − 1] × 1000, respectively. VSMOW is Vienna Standard Mean Ocean Water and VPDB is Vienna Pee Dee Belemnite. The δ^13^C and δ^2^H values of abiotic CH_4_ from Western Tianshan eclogites were determined using a continuous flow isotope ratio mass spectrometry technique with a Thermo Scientific gas chromatograph (GC) and Thermo Finnigan MAT253 isotope ratio mass spectrometer at the Analytical Laboratory, Beijing Research Institute of Uranium Geology, China. Samples of the host minerals (pure garnet or omphacite) were first washed with diluted HCl in order to remove carbonate, and then washed repeatedly with deionized water (DI water) to remove any contaminants from the crystal surfaces. Methane was extracted from the samples by thermally decrepitating the fluid inclusions. This involved heating the samples for 15 min with helium gas at 550°C, passing the released gas through a NaOH trap and a Mg(ClO_4_)_2_ trap to remove CO_2_ and H_2_O, and then through a liquid N_2_ cold trap to enrich CH_4_. Trace amounts of interfering compounds were separated by gas chromatography after pre-concentration of the CH_4_ sample. The purified sample was then either combusted to CO_2_ by reaction with CuO, or pyrolyzed to H_2_ in a silica tube that was heated to 1420°C prior to mass spectrometry. The chromatographic column was PoraPLOT Q (27.5 m × 0.32 mm × 0.10 μm). The δ^13^C and δ^2^H values were corrected by three methane C–H isotope standards, IsoRM 201, 202 and 203 (Qingdao IsotopTech Ltd). The results of the standard samples are consistent with the standard values within error (Supplementary Table S8). The precisions (RSD) of δ^13^C and δ^2^H were better than 0.3‰ and 3‰, respectively. In addition, the continuous flow isotope ratio mass spectrometry technique can quickly carry the released gases out of the heat source and minimize the possible isotopic fractionation. The peaks of the GC spectrum of CH_4_ in both garnet and omphacite are perfect (Supplementary Fig. S10), without any tails, which highlights the good quality of our data.

### Thermodynamic phase equilibrium modeling and oxygen fugacity calculation

The P–T phase diagram (Fig. 4) for the eclogite sample HB142-8 was calculated in the MnNCKFMASCHO system using Perple_X software [53,54] with an internally consistent thermodynamic data set [55]. The details are shown in Supplementary Fig. S9. The Fe^3+^/ΣFe ratio in garnet b, which contained omphacite inclusions in Grt III (Supplementary Fig. S2b), was measured by the flank method [56] with the JEOL JXA-8100 electron microprobe at the Key Laboratory of Orogenic Belts and Crustal Evolution, School of Earth and Space Sciences, Peking University, China. The analytical procedure of the flank method followed reference [57] in detail. The results corresponding to the four zones of garnet (Grt I– IV) are shown in Supplementary Table S3. The oxygen fugacity (*f*O_2_) calculation was calculated for Grt I–IV, and the corresponding omphacite with the equilibrium reaction: 1/3 Ca_3_Al_2_Si_3_O_12_ (garnet) + 5CaFeSi_2_O_6_ (clinopyroxene) + O_2_ = 2Ca_3_Fe_2_Si_3_O_12_ (garnet) + 1/3Fe_3_Al_2_Si_3_O_12_ (garnet) + 4SiO_2_ (coesite) [58]. To relate the results with the P–T conditions obtained with phase equilibrium modeling (Supplementary Fig. S9), we determined *f*O_2_ at 520°C and 22.5 kbar, 550°C and 33 kbar, 570°C and 27.5 kbar and 580°C and 23 kbar, corresponding to Grt I–IV, respectively. Due to the paucity of analyzable omphacite inclusions corresponding to Grt I–II and Grt IV in garnet b, we paired omphacite inclusions from another two garnets, c and d, with Grt I and Grt II, and matrix Omp rim with Grt IV (Supplementary Fig. S3 and Table S1). The compositions of the omphacite inclusions in Grt I–III, and matrix omphacite, are listed in Table S1. The calculated *f*O_2_ are listed in Supplementary Table S4.

### DEW calculation of carbon concentration in fluids

The DEW model [29,30] enables the calculation of reaction equilibrium constants involving minerals, aqueous inorganic and organic ions, complexes, and neutral species. These equilibrium constants combined with the EQ3 fluid speciation code can be used to calculate the aqueous speciation of a fluid in equilibrium with a given mineral assemblage at fixed *f*O_2_, P and T. We selected six points along the P–T–*f*O_2_ trajectory obtained within Fig. 4 to calculate the C-species compositions (Fig. 5 and Supplementary Table S6). Figure 5b–e corresponds to the P–T–*f*O_2_ conditions recorded by Grt I to Grt IV (Fig. 4), except for the pressure: the DEW model was spaced at 5 kbar intervals, and we chose the nearest integer pressure values. The P–T conditions of Fig. 5a and Fig. 5f followed the prograde and retrograde path and the corresponding mineral assemblage deduced in Fig. 4, respectively; *f*O_2_ accorded with reference [25] (450°C, 1.5 GPa, FMQ-1; 550°C, 2.0 GPa, FMQ + 1). The composition of the main solid solutions was set based on the mineral compositions analyzed in the sample. Thus, the molality of carbon in the fluid was not artificially set, but was to be self-consistent based on the dissolved carbon-bearing minerals for the respective metamorphic stages (Fig. 5a–f). The results and input data files are listed in Supplementary Tables S6 and S9. The method used to estimate the abiotic CH_4_ flux is described in Supplementary Note 2.

## Supplementary Material

nwac207_Supplemental_File

## References

[1] Etiope G , Lollar BS. Abiotic methane on earth. Rev Geophys 2013; 51: 276–99. 10.1002/rog.20011

[2] Etiope G , Schoell M. Abiotic gas: atypical, but not rare. Elements 2014; 10: 291–6. 10.2113/gselements.10.4.291

[3] Kelley DS , Karson JA, Früh-Green GL et al. A serpentinite-hosted ecosystem: the lost city hydrothermal field. Science 2005; 307: 1428–34. 10.1126/science.1102556 15746419

[4] Sherwood Lollar B , Lacrampe-Couloume G, Voglesonger K et al. Isotopic signatures of CH_4_ and higher hydrocarbon gases from Precambrian shield sites: a model for abiogenic polymerization of hydrocarbons. Geochim Cosmochim Acta 2008; 72: 4778–95. 10.1016/j.gca.2008.07.004

[5] Proskurowski G , Lilley MD, Seewald JS et al. Abiogenic hydrocarbon production at lost city hydrothermal field. Science 2008; 319: 604–7. 10.1126/science.1151194 18239121

[6] Klein F , Grozeva NG, Seewald JS. Abiotic methane synthesis and serpentinization in olivine-hosted fluid inclusions. Proc Natl Acad Sci USA 2019; 116: 17666–72. 10.1073/pnas.1907871116 31427518 PMC6731755

[7] Kolesnikov A , Kutcherov VG, Goncharov AF. Methane-derived hydrocarbons produced under upper-mantle conditions. Nat Geosci 2009; 2: 566–70. 10.1038/ngeo591

[8] Scott HP , Hemley RJ, Mao H et al. Generation of methane in the earth's mantle: in situ high pressure-temperature measurements of carbonate reduction. Proc Natl Acad Sci USA 2004; 39: 14023–6. 10.1073/pnas.0405930101 PMC521091 15381767

[9] Lobanov SS , Chen PN, Chen XJ et al. Carbon precipitation from heavy hydrocarbon fluid in deep planetary interiors. Nat Commun 2013; 4: 3446. 10.1038/ncomms3446 24026399

[10] Spanu L , Donadio D, Hohl D et al. Stability of hydrocarbons at deep earth pressures and temperatures. Proc Natl Acad Sci USA 2011; 108: 6843–6. 10.1073/pnas.1014804108

[11] Kenney JF , Kutcherov VA, Bendeliani BA et al. A. The evolution of multicomponent systems at high pressures: VI. The thermodynamic stability of the hydrogen–carbon system: the genesis of hydrocarbons and the origin of petroleum. Proc Natl Acad Sci USA 2002; 99: 10976–81. 10.1073/pnas.172376899 12177438 PMC123195

[12] Li Y . Immiscible C-H-O fluids formed at subduction zone conditions. Geochem Persp Lett 2016; 3: 12–21. 10.7185/geochemlet.1702

[13] Huang F , Daniel I, Cardon H et al. Immiscible hydrocarbon fluids in the deep carbon cycle. Nat Commun 2017; 8:15798. 10.1038/ncomms15798 28604740 PMC5472781

[14] Mukhina E , Kolesnikov A, Kutcherov V. The lower pT limit of deep hydrocarbon synthesis by CaCO_3_ aqueous reduction. Sci Rep 2017; 7: 5749. 10.1038/s41598-017-06155-6 28720804 PMC5515916

[15] Vitale Brovarone A , Martinez I, Elmaleh A et al. Massive production of abiotic methane during subduction evidenced in metamorphosed ophicarbonates from the Italian Alps. Nat Commun 2017; 8: 14134. 10.1038/ncomms14134 28223715 PMC5322563

[16] Vitale Brovarone A , Sverjensky DA, Piccoli F et al. Subduction hides high-pressure sources of energy that may feed the deep subsurface biosphere. Nat Commun 2020; 11:3880. 10.1038/s41467-020-17342-x 32759942 PMC7406650

[17] Peng WG , Zhang LF, Tumiati S et al. Abiotic methane generation through reduction of serpentinite-hosted dolomite: implications for carbon mobility in subduction zones. Geochim Cosmochim Acta 2021; 311: 119–40. 10.1016/j.gca.2021.07.033

[18] Li SG , Yang W, Ke S et al. Deep carbon cycles constrained by a large-scale mantle Mg isotope anomaly in eastern China. Natl Sci Rev 2017; 4: 111–20. 10.1093/nsr/nww070

[19] Kelemen PB , Manning CE. Reevaluating carbon fluxes in subduction zones, what goes down, mostly comes up. Proc Natl Acad Sci USA 2015; 112: 3997–4006. 10.1073/pnas.1507889112 26048906 PMC4522802

[20] Plank T , Manning CE. Subducting carbon. Nature 2019; 574: 343–52. 10.1038/s41586-019-1643-z 31619791

[21] Stewart EM , Ague JJ. Pervasive subduction zone devolatilization recycles CO_2_ into the forearc. Nat Commun 2020; 11: 6220. 10.1038/s41467-020-19993-2 33277477 PMC7718257

[22] Chen CF , Förster MW, Foley SF et al. Massive carbon storage in convergent margins. Nat Commun 2021; 12: 4463. 10.1038/s41467-021-24750-0 34294696 PMC8298627

[23] Dasgupta R , Hirschmann MM. The deep carbon cycle and melting in Earth's interior. Earth Planet Sci Lett 2010; 298: 1–13. 10.1016/j.epsl.2010.06.039

[24] Zhang LF , Wang Y, Zhang LJ et al. Ultrahigh pressure metamorphism and tectonic evolution of southwestern Tianshan orogenic belt, China: a comprehensive review. Geol Soc Spec Publ 2019; 474: 133–52. 10.1144/SP474.12

[25] Tao RB , Zhang LF, Tian M et al. Formation of abiotic hydrocarbon from reduction of carbonate in subduction zones: constraints from petrological observation and experimental simulation. Geochim Cosmochim Acta 2018; 239: 390–408. 10.1016/j.gca.2018.08.008

[26] Grozeva MG , Klein F, Seewald JS et al. Chemical and isotopic analyses of hydrocarbon-bearing fluid inclusions in olivine-rich rocks. Phil Trans R Soc A 2020; 378: 20180431. 10.1098/rsta.2018.0431 31902341 PMC7015310

[27] Reeves EP , Fiebig J. Abiotic synthesis of methane and organic compounds in earth's lithosphere. Elements 2020; 16: 25–31. 10.2138/gselements.16.1.25

[28] Rahl JM , Anderson KM, Brandon MT et al. Raman spectroscopic carbonaceous material thermometry of low-grade metamorphic rocks: calibration and application to tectonic exhumation in Crete, Greece. Earth Planet Sci Lett 2005; 240: 339–54. 10.1016/j.epsl.2005.09.055

[29] Sverjensky DA , Harrison B, Azzolini D. Water in the deep Earth: the dielectric constant and the solubilities of quartz and corundum to 60 kb and 1200°C. Geochim Cosmochim Acta 2014; 129: 125–45. 10.1016/j.gca.2013.12.019

[30] Huang F , Sverjensky DA. Extended deep earth water model for predicting major element mantle metasomatism. Geochim Cosmochim Acta 2019; 254: 192–230. 10.1016/j.gca.2019.03.027

[31] Li JL , Schwarzenbach EM, John T et al. Uncovering and quantifying the subduction zone sulfur cycle from the slab perspective. Nat Commun 2020; 11: 514. 10.1038/s41467-019-14110-4 31980597 PMC6981181

[32] Piccoli F , Hermann J, Pettke T et al. Subducting serpentinites release reduced, not oxidized, aqueous fluids. Sci Rep 2019; 9: 19573. 10.1038/s41598-019-55944-8 31862932 PMC6925189

[33] Gerrits AR , Inglis EC, Dragovic B et al. Release of oxidizing fluids in subduction zones recorded by iron isotope zonation in garnet. Nat Geosci 2019; 12: 1029–33. 10.1038/s41561-019-0471-y

[34] Ague JJ , Nicolescu S. Carbon dioxide released from subduction zones by fluid-mediated reactions. Nat Geosci 2014; 7: 355–60. 10.1038/ngeo2143

[35] Frezzotti ML , Selverstone J, Sharp ZD et al. Carbonate dissolution during subduction revealed by diamond-bearing rocks from the Alps. Nat Geosci 2011; 4: 703–6. 10.1038/ngeo1246

[36] Cannaò E , Malaspina N. From oceanic to continental subduction: implications for the geochemical and redox evolution of the supra–subduction mantle. Geosphere 2018; 14: 2311–36. 10.1130/GES01597.1

[37] Tumiati S , Malaspina N. Redox processes and the role of carbon-bearing volatiles from the slab–mantle interface to the mantle wedge. J Geol Soc London 2019; 176: 388–97. 10.1144/jgs2018-046

[38] Tumiati S , Recchia S, Remusat L et al. Silicate dissolution boosts the CO_2_ concentrations in subduction fluids. Nat Commun 2017; 8: 616. 10.1038/s41467-017-00562-z PMC5606994 28931819

[39] Peña-Alvarez M , Vitale Brovarone A, Donnelly ME et al. *In-situ* abiogenic methane synthesis from diamond and graphite under geologically relevant conditions. Nat Commun 2021; 12: 6387. 10.1038/s41467-021-26664-3 34737292 PMC8569197

[40] Horibe Y , Craig H. D/H fractionation in the system methane-hydrogen-water. Geochim Cosmochim Acta 1995; 59: 5209–17. 10.1016/0016-7037(95)00391-6

[41] Shaw AM , Hauri EH, Fischer TP et al. Hydrogen isotopes in mariana arc melt inclusions: implications for subduction dehydration and the deep-earth water cycle. Earth Planet Sci Lett 2008; 275: 138–45. 10.1016/j.epsl.2008.08.015

[42] Boutier A , Vitale Brovarone A, Martinez I et al. High pressure serpentinization and abiotic methane formation in metaperidotite from the Appalachian subduction, northern Vermont. Lithos 2021; 396: 106190. 10.1016/j.lithos.2021.106190

[43] Song SG , Su L, Niu YL et al. CH_4_ inclusions in orogenic harzburgite: evidence for reduced slab fluids and implication for redox melting in mantle wedge. Geochim Cosmochim Acta 2009; 73: 1737–54. 10.1016/j.gca.2008.12.008

[44] Fu B , Touret J, Zheng YF. Remnants of premetamorphic fluid and oxygen isotopic signatures in eclogites and garnet clinopyroxenite from the Dabie-Suluterranes, eastern China. J Metamorph Geol 2003; 21: 561–78. 10.1046/j.1525-1314.2003.00464.x

[45] Mukherjee BK , Sachan HK. Fluids in coesite-bearing rocks of the Tso Moraricomplex, NW Himalaya: evidence for entrapment during peak metamorphism and subsequent uplift. Geol Mag 2009; 146: 876–89. 10.1017/S0016756809990069

[46] van Keken PE , Hacker BR, Syracuse EM et al. Subduction factory: 4. Depth-dependent flux of H_2_O from subducting slabs worldwide. J Geophys Res 2011; 116: B01401. 10.1029/2010JB007922

[47] Abers GA , van Keken PE, Hacker BR. The cold and relatively dry nature of mantle forearcs in subduction zones. Nat Geosci 2017; 10: 333–7. 10.1038/ngeo2922

[48] Sverjensky DA , Stagno V, Huang F. Important role for organic carbon in subduction-zone fluids in the deep carbon cycle. Nat Geosci 2014; 7: 909–13. 10.1038/ngeo2291

[49] Evans KA . The redox budget of subduction zones. Earth-Sci Rev 2012; 113: 11–32. 10.1016/j.earscirev.2012.03.003

[50] Zhang C , Duan Z. A model for C–O–H fluid in the Earth'smantle. Geochim Cosmochim Acta 2009; 73: 2089–102. 10.1016/j.gca.2009.01.021

[51] Schmidt MW , Poli S. Devolatilization during subduction. In: Holland HD (ed.). Treatise on Geochemistry. New York: Elsevier Science Ltd, 2014, 4.19, 669–701.

[52] Merdith AS , del Real PG, Daniel I et al. Pulsated global hydrogen and methane flux at mid-ocean ridges driven by Pangea breakup. Geochem Geophys Geosyst 2020; 21: e2019GC008869. 10.1029/2019GC008869

[53] Connolly JAD . Multivariable phase-diagrams: an algorithm based on generalized thermodynamics. Am J Sci 1990; 290: 666–718. 10.2475/ajs.290.6.666

[54] Connolly JAD . Computation of phase equilibria by linear programming: a tool for geodynamic modelling and its application to subduction zone decarbonation. Earth Planet Sci Lett 2005; 236: 524–41. 10.1016/j.epsl.2005.04.033

[55] Holland TJB , Powell R. An internally consistent thermodynamic data set for phases of petrological interest. J Metamorph Geol 1998; 16: 309–43. 10.1111/j.1525-1314.1998.00140.x

[56] Höfer HE , Brey GP. The iron oxidation state of garnet by electron microprobe: its determination with the flank method combined with major-element analysis. Am Mineral 2007; 92: 873–85. 10.2138/am.2007.2390

[57] Li XL , Song SG, Zhang LF et al. Application of microprobe-based flank method analysis of F e^3+^ in garnet of North Qilian eclogite and its geological implication. Sci Bull 2018; 63: 300–5. 10.1016/j.scib.2018.01.025

[58] Stagno V , Frost DJ, McCammon CA et al. The oxygen fugacity at which graphite or diamond forms from carbonate-bearing melts in eclogitic rocks. Contrib Miner Petrol 2015; 169: 16. 10.1038/nature11679

