# Supplementary material for: Massive abiotic methane production in eclogite during cold subduction
Source: Natl Sci Rev. 2022 Sep 30;10(1):nwac207. doi: 10.1093/nsr/nwac207 (PMC9840456; doi:10.1093/nsr/nwac207)
Supplement: nwac207_Supplemental_File [file nwac207_supplemental_file.pdf]

Massive abiotic methane production in eclogite during cold subduction

Lijuan Zhang, Lifei Zhang*, Ming Tang, Xiao Wang, Renbiao Tao, Cheng Xu, and Thomas Bader

Key Laboratory of Orogenic Belts and Crustal Evolution, MOE, School of Earth and Space Sciences, Peking University, Beijing 100871, China

*Corresponding author: Lifei Zhang

Email: lfzhang@pku.edu.cn

Supplementary Information

This PDF file includes:

Supplementary note 1-2
Figs. S1 to S10
Tables S1 to S9
References

Supplementary Note 1

Ankeritic dolomite and CO_2-rich fluid inclusions. Two types of ankeritic dolomite (Fe-Dol) occur in the studied eclogite (Fig. S7). Type 1 is rare, residual, prograde, porphyritic dolomite in contact with omphacite (Fig. S7a); it has $Fe^{\#}$ [$=Fe^{2+}/(Mg+Fe^{2+})$] of 0.29-0.25 (Table S1). Microtextures imply the decomposition of Type 1 Fe-Dol to aragonite/calcite, magnesite, magnetite, and graphite (Fig. S7a). Residual aragonite was identified by Raman spectroscopy (Fig. S7b). Type 2 Fe-Dol coexists with amphibole, epidote, titanite, and paragonite, and belongs to the epidote-amphibolite facies retrograde mineral assemblage (Fig. S7c-e). Back scattered electron images highlight the $Fe^{\#}$ zoning in Type 2 Fe-Dol: $Fe^{\#}$ is 0.18 to 0.16 in the brighter core, but 0.15-0.13 in the darker rim (Fig. S7e). The cores commonly contain residual magnesite and calcite pseudomorphs after aragonite (Fig. S7c-e).

The CO_2-rich fluid inclusions were only observed in the type 2 Fe-Dol, in both core and rim. The CO_2-rich fluid inclusions are mostly larger than the CH_4-rich fluid inclusions in garnet, commonly 2-10 μm in size (Figs. S7f-g and S8). Their shape is also more regular, mostly short or long columnar, and ellipsoid (Fig. S7f-g and S8). The CO_2-rich fluid inclusions contain liquid + vapor at room temperature. The ubiquitous CO_2 vapor has sharp Raman peaks at ~1285 and ~1388 cm^{-1}, and weak bands from 2331 to 2328 cm^{-1} reveal N_2. The fluid phase has the characteristic broad Raman peak for H_2O at 3440 cm^{-1} (Fig. S8). Because the rim of type 2 Fe-Dol belongs to the epidote-amphibolite facies stage, and thus we can conclude that the CO_2-rich fluid inclusions were entrapped at this stage during exhumation. It is worth pointing out that both CO_2 and CH_4 coexist in the vapor phase of several fluid inclusions in the core of type 2 Fe-Dol (Fig. S8h), which occurs besides a magnesite remnant of the peak metamorphism. The coexistence of CO_2 and CH_4 is revealed by Raman peaks at 1286, 1388, and 2917 cm^{-1}; the fluid phase yields the peak of H_2O at 3440 cm^{-1} (Fig. S8h). The peaks of CO_2 are stronger than those of CH_4, indicating CO_2 is the dominating carbon species.

The omnipresent N_2, whether in CH_4-rich (Fig. 2a-d, Fig S5-S6) or CO_2-rich (Fig. 2e, Fig S8) fluid inclusions in our UHP eclogites, indeed exists in the inclusions. During

Raman spectroscopy analysis, we checked the effect of atmospheric N_2 contamination with the equivalent analytical protocol on the host minerals (garnet, omphacite and dolomite) and it appeared negligible. There are no indications for N_2 in the Raman spectra of "Grt BG", "Omp BG" and "Dol BG" in Figs. 2, S5, S6, and S8. Fluids released by the dehydration of subducted sediments in the process of HP-UHP metamorphism may be the source of the N_2 observed in our samples, because subducting slabs, especially subducted sedimentary rocks, host orders of magnitude more nitrogen than mantle rocks (Bebout et al, 2013).

Supplementary Note 2

The method to calculate the abiotic CH_4 flux. The CH_4 flux can be estimated from the known amount of CH_4 in per kg of H_2O [mole CH_4/kg H_2O], multiplied by the amount of H_2O that was released from the subducted slab [percent H_2O], and the total mass of eclogites subducted annually [weight of total eclogites subducted annually]. The formula can be written as:

CH_4 flux = [mole CH_4/kg H_2O] × [percent H_2O] × [weight of total eclogites subducted annually]

The calculation procedures for each item are described below:

1. [mole CH_4/kg H_2O]: We can obtain the mole concentration of CH_4 per kg of H_2O (i.e., molar conc in Table S6) based on our DEW model of the prograde subduction metamorphism at specific P-T-fO_2 conditions (Fig. 5a-c). At the conditions of Fig. 5a (450°C, 1.5 GPa, FMQ-1), Fig. 5b (510°C, 2.5 GPa, FMQ-2.4), and Fig. 5c (550°C, 3.5 GPa, FMQ-3.5), the molar concentrations of CH_4 per kg of H_2O in the released fluid are 4.61, 0.938, and 1.512, respectively (Table S6). Thus, the average CH_4 content is 2.353 mole/kg H_2O.
2. [percent H_2O]: We estimated the proportion of H_2O with the data for MORB (the protolith rock of eclogite) from Schmidt and Poli, (2014): the H_2O content

in MORB at the conditions of the prograde blueschist to eclogite facies at ~50 km (Fig. 5a: 450°C, 1.5 GPa) is ~5.0 wt%, and at the conditions of UHP eclogite facies at ~120 km (Fig. 5c: 550°C, 3.5 GPa,) is ~0.8 wt%. Thus, the H_2O released from MORB is 5-0.8=4.2 wt%, which we rounded to ~4 wt% for a more conservative estimate.

3. [weight of total eclogites subducted annually]:

(1) For the estimates of the modern subduction zones worldwide, all parameters of eclogite are from Kelemen and Manning (2015), i.e., the total length of the subduction zones worldwide on Earth is 44454 km, the thickness of the eclogites is 1000 m, the subduction rate is 0.054 m/y, and the density of eclogite is 3000 kg/m^3 (Table S7). Consequently, the multiplication of 44454000 m × 1000 m × 0.054 m/y × 3000 kg/m^3 yields 7.2×10^{12} kg/y for the total amount of eclogites subducted annually around the world.

(2) For the estimates of the Southern Tianshan and the global cases of cold and reduced paleo-subduction zones, except for the length of the subduction zone, the parameters are the same as described above.

i. The Western Tianshan HP-UHP belt extends ~2000 km from the eastern Kumishi buleschist belt westwards to the Atabishi eclogite belt of Kyrgyzstan and the Fan-Karategin HP-UHP metamorphic belt. They have similar protolith compositions and P-T-fO_2 conditions, and together form the Southern Tianshan cold paleo-subduction zone (Zhang et al., 2018). Thus, the length of the Southern Tianshan cold paleo-subduction zone is 2000 km, i.e., 2000000 m in Table S7. Consequently, the multiplication of 2000000 m × 1000 m × 0.054 m/y × 3000 kg/m^3 yields 3.24×10^{11} kg/y for the eclogites subducted annually in the Southern Tianshan.

ii. The total length of all the global cold subduction zones that are reduced enough to produce abiotic CH_4 is unknown, because of the limited knowledge about abiotic CH_4 formation in eclogites during subduction. Only considering the eclogites capable of producing abiotic CH_4 in the cold

paleo-subduction zones of the Alps (~1200 km), Appalachians (~3200km), Southern Tianshan (~2000 km), and Qilian (~800km), where abiotic CH_4 has been reported (Vitale Brovarone et al., 2017; 2020; Peng et al., 2021; Boutier et al., 2021; Song et al., 2009; Tao et al., 2018), results in a total length of 1200 + 3200 + 2000 + 800 = 7200km. Consequently, the multiplication of 7200000 m × 1000 m × 0.054 m/y × 3000 kg/m^3 yields 1.17×10^{12} kg/y for the eclogites subducted annually (Table S7).

4. With the above CH_4 flux formula, the data yield: (1) for the modern subduction zones worldwide, CH_4 flux = 2.353 mol/kg H_2O × 4 wt% × 7.2×10^{12} kg/y = 6.78×10^{11} mol/y, i.e., 10.8 Mt/y CH_4, corresponding to 8.14 Mt/y of carbon after unit conversion; (2) for the ancient Southern Tianshan, CH_4 flux = 2.353 mol/kg H_2O × 4 wt% × 3.24×10^{11} kg/y=3.05×10^9 mol/y, i.e., 0.49 Mt/y, equal to 0.37 Mt/y of carbon; (3) for the cases of cold and reduced paleo-subduction zones, CH_4 flux = 2.353 mol/kg H_2O × 4 wt% × 1.17×10^{12} kg/y = 1.1×10^{11} mol/y, i.e., 1.76 Mt/y CH_4, corresponding to 1.32 Mt/y carbon (Table S7).

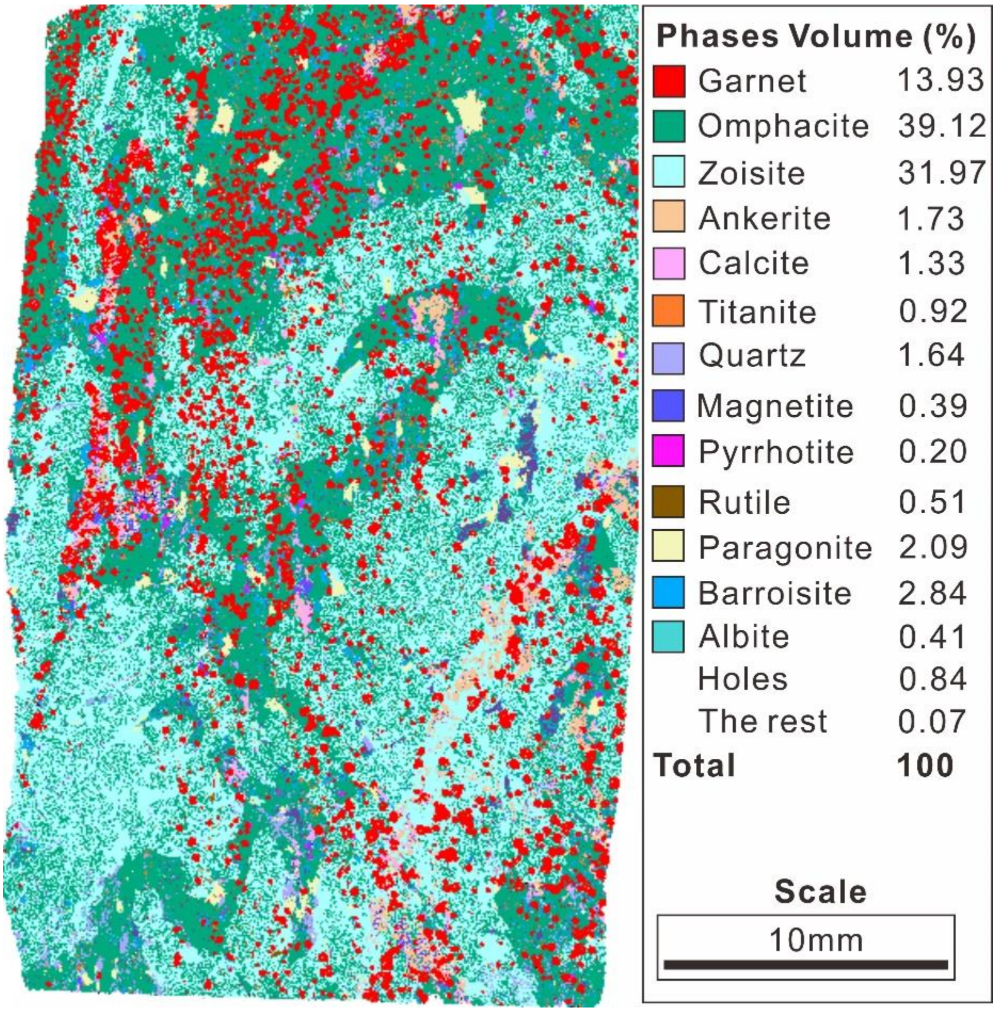

Fig. S1. Mineral map of a representative thin section of the CH_4-rich eclogite sample HB142-8**.** The eclogite contains typical minerals of garnet (Grt) and omphacite (Omp), with a few ankerite (Fe-Dol), and shows a little retrogressed to zoisite (Zo), barroisite (Bar), paragonite (Pa), albite (Ab) and titanite (Ttn).

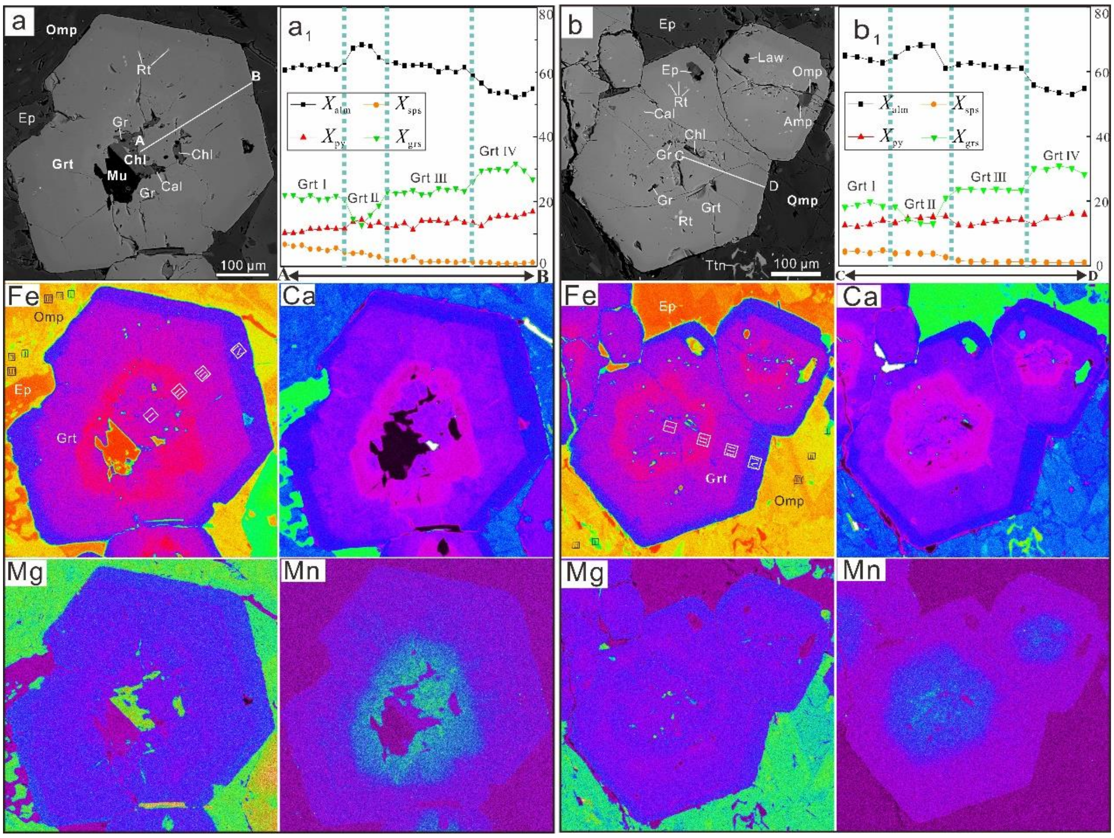

Fig. S2. BSE images showing the mineral assemblages and inclusions enclosed in two representative garnets. Major element composition profiles correspond to A-B and C-D in garnets a and b. X_{alm} =Fe^{2+} / (Ca + Mg + Fe^{2+} + Mn) × 100, X_{grs} =Ca / (Ca + Mg + Fe^{2+} + Mn) ×100, X_{py} =Mg / (Ca + Mg + Fe^{2+} + Mn) × 100 and X_{sps} =Mn / (Ca + Mg + Fe^{2+} + Mn) × 100. The colorized X-ray maps reveal the four growth zones of garnet (Grt I to Grt IV) in iron (Fe), calcium (Ca), magnesium (Mg) and manganese (Mn).

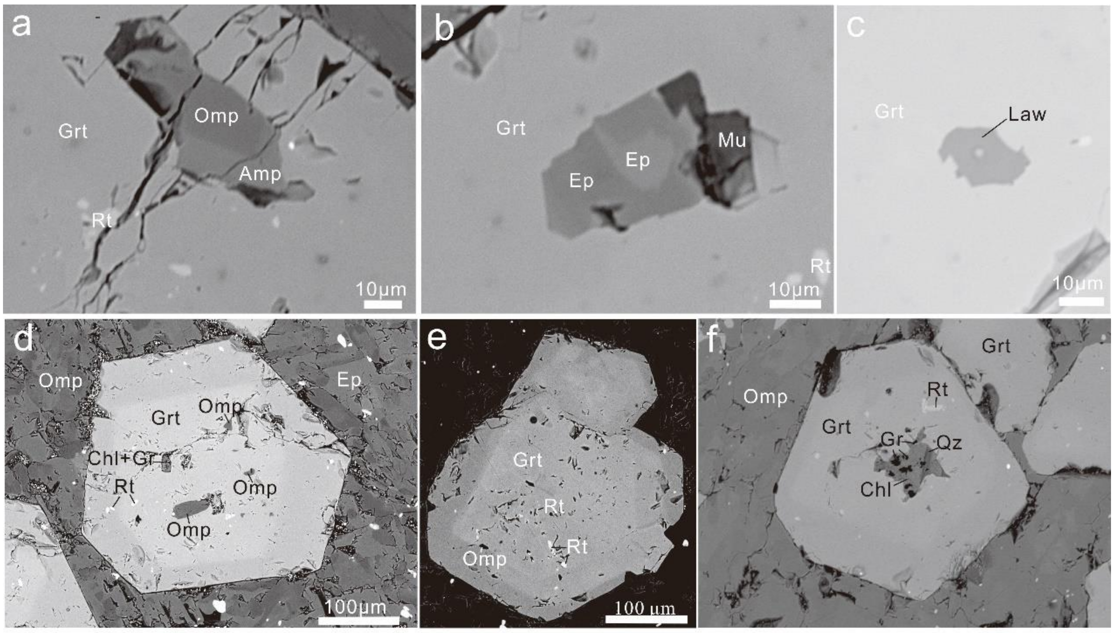

Fig. S3. BSE images showing the mineral inclusions enclosed in garnets. a-c, Mineral inclusions enclosed in garnet b in Fig. S2. a, Omphacite inclusion in Grt III, which was used to calculate the fO_2. b, Epidote and muscovite inclusions in Grt IV. c, Lawsonite inclusion in Grt III. d, Three omphacite inclusions in Grt I in a third garnet c. e, Omphacite inclusion in Grt II of a fourth garnet d. The omphacite inclusions in garnets c and d were used to calculate the fO_2 with the corresponding compositions of Grt I and Grt II of garnet b in Fig. S2 measured with the flank method. f, Rutile, chlorite and graphite inclusions commonly and quartz occasionally occur in Grt I of a fifth garnet e.

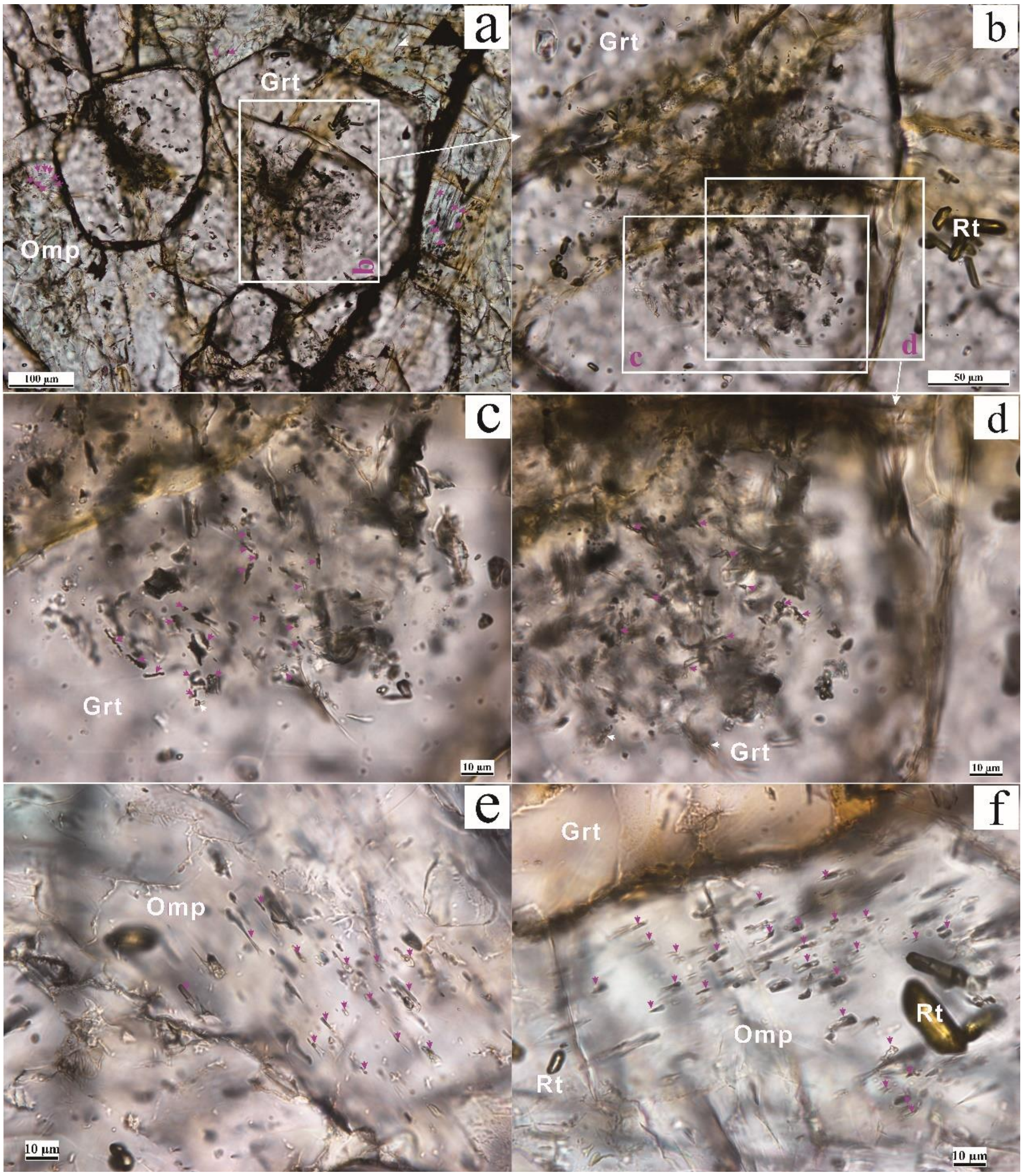

Fig. S4. Photomicrographs of CH_4-rich fluid inclusions in garnet (Grt) and omphacite (Omp). a, Both garnet and omphacite contain CH_4-rich fluid inclusions (purple arrows). The garnet is the one featured in Fig.1a and Fig. S2b. b, Enlargement of the CH_4-rich fluid inclusions in Grt I-II. c-d, Enlargements of b showing the distributions, morphologies, and sizes of the CH_4-rich fluid inclusions (purple arrows). The growth of the fluid inclusions along the garnet zonation, indicates fluid entrapment during garnet crystallization. e-f, Primary fluid inclusions (purple arrows) in omphacite occur as isolated parallel tubes or as non-planar clusters, and their long expansions are parallel to the c-axes of the host omphacites.

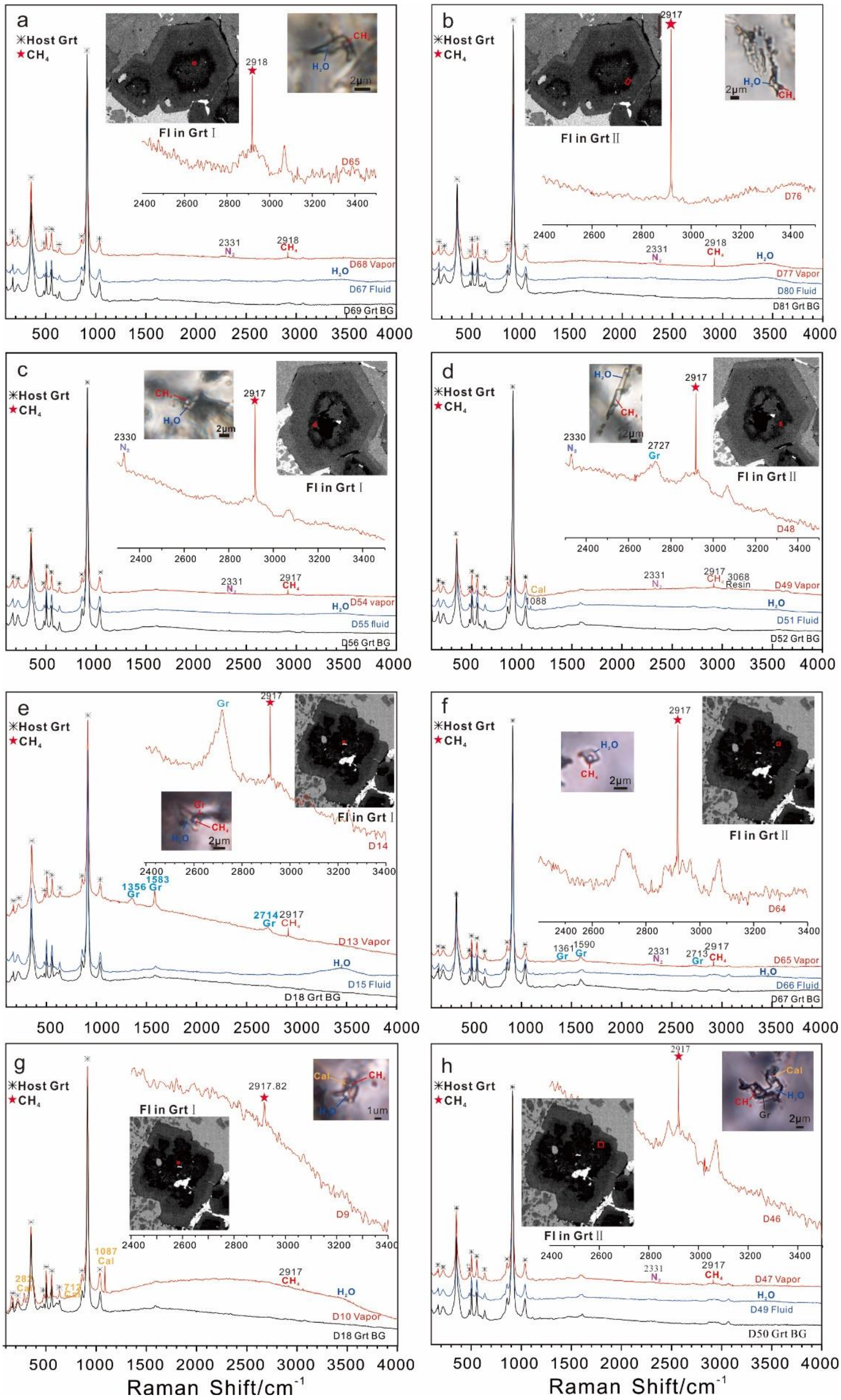

Fig. S5. Representative Raman spectra of CH_4-rich fluid inclusions in garnets. "Grt BG" represents the background peaks of the host garnets. The inset Raman spectra are retest that confirm the existences of CH_4. The inset images show the locations and the morphologies of the CH_4-rich fluid inclusions (FIs).

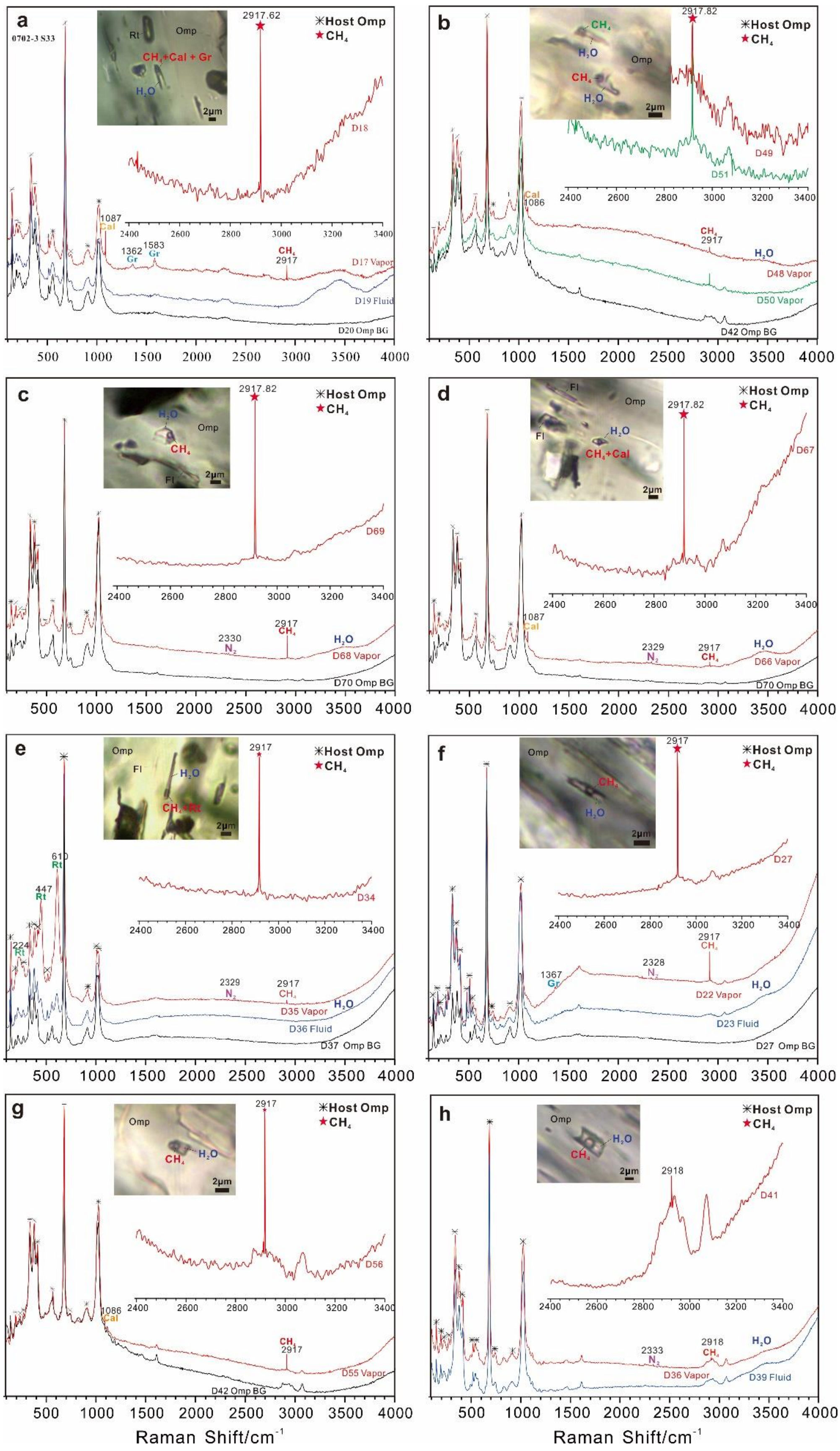

Fig. S6. Representative Raman spectra of CH_4-rich fluid inclusions in omphacite. "Omp BG" indicates the background peaks of the host omphacite. The inset Raman spectra are retests that confirm the existence of CH_4. The inset images show the corresponding CH_4-rich fluid inclusions.

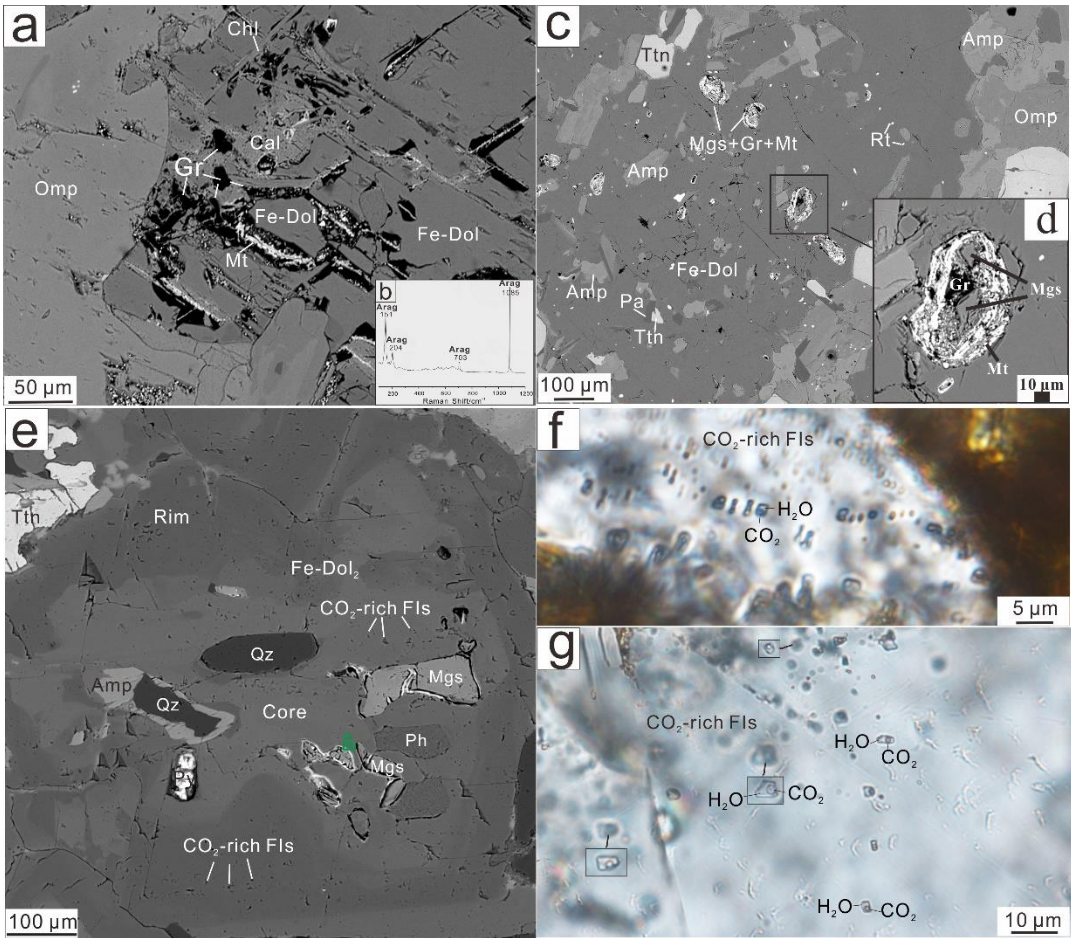

Fig. S7. BSE images of the carbonated eclogite (HB142-8). a, Type 1 denotes rare residual porphyritic ankeritic dolomite (Fe-Dol) decomposed to aragonite (Arag)/calcite (Cal), magnesite (Mgs), magnetite (Mt), and graphite (Gr). b, Raman spectrum of residual Arag in Fe-Dol. c, Type 2 ankeritic dolomite coexists with amphibole, epidote, titanite, and paragonite, belongs to the epidote-amphibolite facies retrograde mineral assemblage, and shows a few residual aggregations of Mgs + Mt + Gr in the core. d, Enlargement of the residual aggregations of Mgs + Mt + Gr. e, CO_2-rich fluid inclusions observed in both core and rim of type 2 Fe-Dol, and residual compounds of Mgs + phengite (Ph) in the core. f-g, examples of CO_2-rich fluid inclusions in the core (f) and in the rim (g) of type 2 Fe-Dol, with a gas phase of CO_2 and a liquid phase of H_2O.

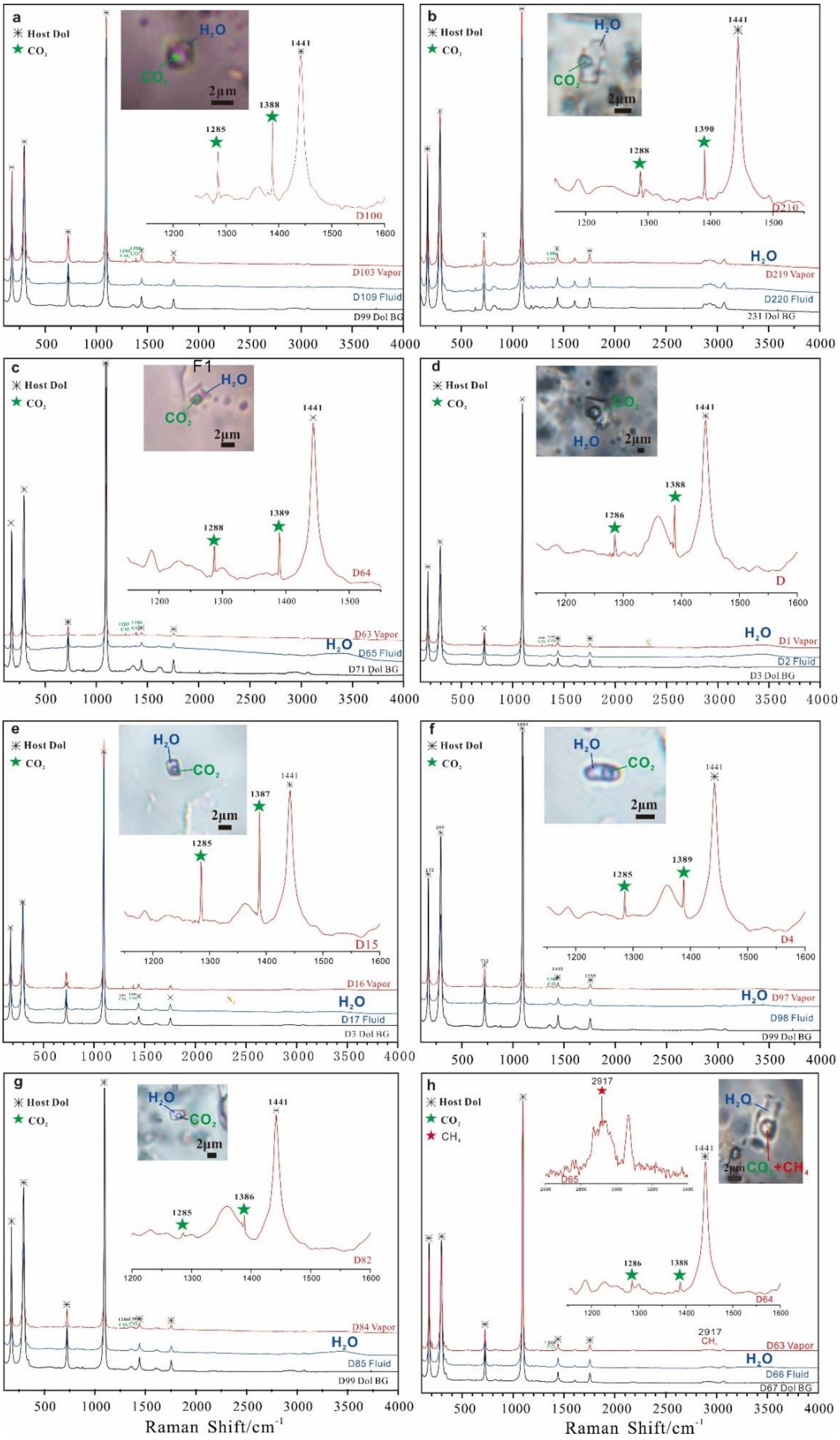

Fig. S8. Representative Raman spectra of CO_2-rich FIs in Fe-dolomite. “Dol BG” indicates the background peaks of the host dolomite. The inset Raman spectra are retests that confirm the existence of CO_2. The inset images show the corresponding CO_2-rich fluid inclusions.

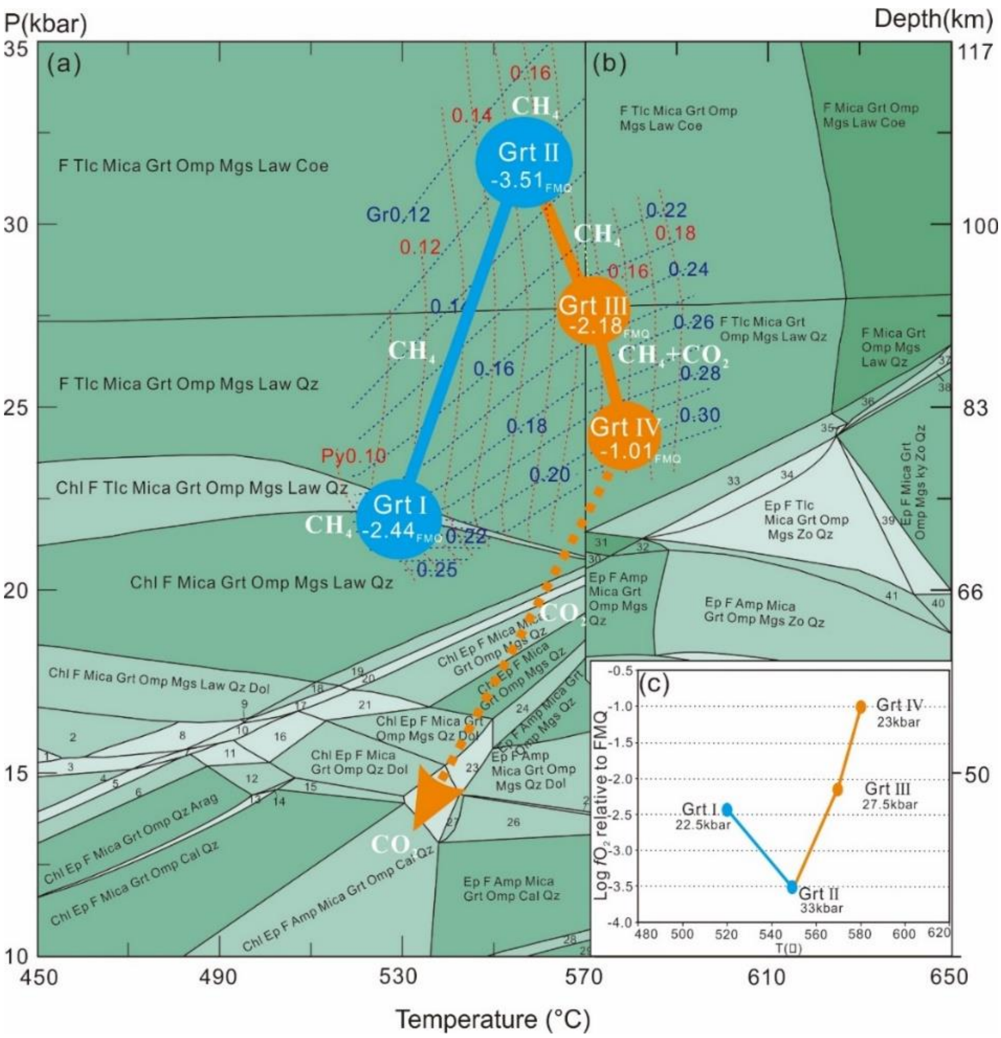

Fig. S9. P–T pseudosection of the representative CH_4-rich eclogite sample HB142-8, calculated in the system MnNCKFMASCHO with the effective bulk composition (provided in Table S5) for prograde (a) and retrograde (b) metamorphism using Perple_X. The following solid solution models were adopted: garnet (White et al., 2007), omphacite (Diener and Powell, 2012), amphibole (Dale et al., 2005), white mica (Coggon and Holland, 2002), chlorite (Holland and Powell, 1998), epidote (Holland and Powell, 1998), ternary Ca–Fe–Mg carbonates (Franzolin et al., 2011), and H_2O–CO_2 (Connolly and Trommsdorff, 1991). P–T conditions corresponding to the garnet compositional profiles are plotted (Fig. S2 and Table S1). Isopleths of the grossular (Ca/[Ca + Mg + Mn + Fe^{2+}]) and the pyrope component (Mg/[Ca + Mg + Mn + Fe^{2+}]) are shown as dotted blue and red lines, respectively. The further retrograde evolution is inferred from the late growth of amphibole and chlorite, and the generalized P–T trajectory is shown by arrow. (c) The oxygen fugacity (fO_2) results were constrained by pairs of garnet and corresponding omphacite (Table S1 and Table S3).

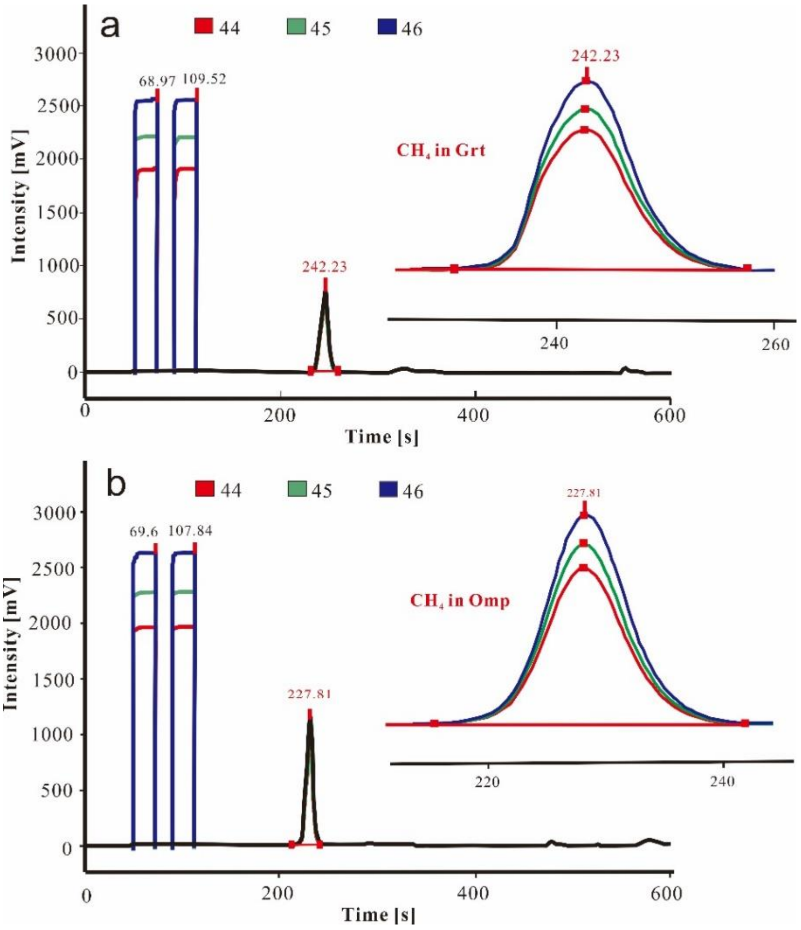

Fig. S10. Representative gas chromatograms of the gases extracted by decrepitation from the eclogite samples. a, the peak of gas released from pure garnet. b, the peak of gas released from pure omphacite. The insert figures enlarge the peaks of CH_4 released from garnet and omphacite, which are without any tails, indicating the good quality of our data.

Table S1. The major element compositions of minerals in the representative CH_4-rich eclogite

Mineral	Grt Ⅰ	Grt Ⅱ	Grt Ⅲ	Grt Ⅳ	Grt Ⅰ	Grt Ⅱ	Grt Ⅲ	Grt Ⅳ
	Grt a				Grt b			
SiO_2	37.79	37.75	37.89	38.03	37.69	37.71	37.51	38.64
TiO_2	---	0.11	0.12	0.03	0.08	0.05	0.03	0.05
Al_2O_3	21.25	20.86	21.35	21.65	21.43	20.80	21.23	21.50
Cr_2O_3	---	0.02	0.02	0.02	0.07	0.06	0.04	---
FeO	27.98	32.32	28.07	24.44	28.67	32.15	28.12	24.49
MnO	3.14	1.41	0.88	0.42	2.41	1.20	1.04	0.47
MgO	3.33	3.52	3.61	3.98	3.55	3.40	3.31	3.80
CaO	6.60	4.41	7.99	10.50	6.49	4.23	8.02	11.23
Na_2O	0.08	0.03	---	0.02	0.01	0.05	0.04	0.02
K_2O	---	---	---	---	---	---	0.01	0.01
Totals	100.17	100.43	99.93	99.09	100.4	99.65	99.35	100.21
O	12	12	12	12	12	12	12	12
Si	2.991	2.999	2.991	2.993	2.975	3.019	2.983	3.011
Ti	---	0.007	0.007	0.002	0.005	0.003	0.002	0.003
Al	1.983	1.954	1.987	2.009	1.994	1.963	1.990	1.975
Cr	---	0.001	0.001	0.001	0.004	0.004	0.003	---
Fe^{3+}	0.049	0.038	0.014	0.003	0.044	---	0.046	---
Fe^{2+}	1.803	2.109	1.839	1.609	1.849	2.152	1.824	1.596
Mn	0.210	0.095	0.059	0.028	0.161	0.081	0.070	0.031
Mg	0.393	0.417	0.425	0.467	0.418	0.406	0.392	0.441
Ca	0.560	0.375	0.676	0.886	0.549	0.363	0.683	0.938
Na	0.012	0.005	0.005	0.003	0.002	0.008	0.006	0.003
K	---	---	---	---	---	---	0.001	0.001
Sum	8	8	8	8	8	8	8	8
X_{alm}	60.79	70.39	61.32	53.81	62.11	71.69	61.43	53.09
X_{sps}	7.08	3.17	1.97	0.94	5.41	2.70	2.36	1.03
X_{py}	13.25	13.92	14.17	15.62	14.04	13.52	13.20	14.67
X_{grs}	18.88	12.52	22.54	29.63	18.44	12.09	23.00	31.20

Table S1: continued

Mineral	Omp	Omp	Omp	Omp C	Omp M	Omp R
Location	In Grt I	In Grt II	In Grt III	In M	In M	In M
SiO_2	55.25	57.19	56.46	56.1	56.19	57.05
TiO_2	0.08	---	---	0.10	0.04	0.01
Al_2O_3	8.53	10.42	10.67	8.37	9.95	11.22
Cr_2O_3	0.04	0.06	0.04	0.08	0.04	0.20
FeO	6.98	5.00	4.65	7.77	5.73	2.85
MnO	0.03	0.08	0.08	0.08	0.06	0.06
MgO	8.33	8.45	8.49	7.51	7.68	9.06
CaO	13.96	13.43	13.44	12.78	13.19	13.43
Na_2O	6.27	6.48	6.40	6.76	7.03	6.32
K_2O	---	---	---	---	---	0.01
Totals	99.47	101.18	100.23	99.55	99.91	100.21
O	6	6	6	6		6
Si	1.995	2.013	2.004	2.028	2.006	2.006
Ti	0.002	0.002	---	0.003	0.001	---
Al	0.363	0.432	0.446	0.357	0.419	0.465
Cr	0.001	0.002	0.001	0.002	0.001	0.006
Fe^{3+}	0.081	---	---	0.054	0.052	---
Fe^{2+}	0.130	0.147	0.138	0.181	0.119	0.084
Mn	0.001	0.002	0.002	0.002	0.002	0.002
Mg	0.448	0.443	0.449	0.405	0.409	0.475
Ca	0.540	0.506	0.511	0.495	0.505	0.506
Na	0.439	0.442	0.440	0.474	0.487	0.431
K	---	---	---	---	---	---
Sum	4	4	4	4	4	4
Jd	35.8	44.5	45.0	38.5	42.5	47.1
Ae	8.1	0	0	5.4	5.2	0
WEE	56.1	55.5	55.0	56.1	52.3	52.9

Table S1: continued

Mineral	Mg-Cal	Mg-Cal	Cal	Dol	Dol C	Dol R	Mgs	Mgs
Location	In Grt	In Dol	Ar Dol	Type 1	Type 2		In Dol	In Dol
SiO_2	0.09	0.04	0.05	0.01	0.02	0.02	---	---
TiO_2	0.01	---	0.03	0.01	0.01	---	---	0.05
Al_2O_3	0.04	0.02	0.01	---	---	0.04	---	---
Cr_2O_3	---	0.01	---	0.03	---	---	0.07	---
FeO	1.99	2.07	0.76	10.04	6.82	4.98	27.98	26.99
MnO	0.71	0.19	0.17	0.23	0.09	---	0.12	0.08
MgO	0.86	1.47	0.39	15.76	17.99	18.04	26.5	27.53
CaO	57.29	56.01	58.19	28.48	29.42	28.61	0.27	0.39
Na_2O	---	---	0.03	---	---	0.04	0.02	0.02
K_2O	---	---	---	---	0.01	---	---	---
Totals	60.99	59.81	59.63	54.56	54.36	51.74	54.96	55.06
O	0	0	0	0	0	0	0	0
Si	0.003	0.001	0.002	---	0.001	0.001	---	---
Ti	---	---	0.001	---	---	---	---	0.001
Al	0.001	0.001	---	---	---	0.002	---	---
Cr	---	---	---	0.001	---	---	0.002	---
Fe^{3+}	---	---	---	---	---	---	---	---
Fe^{2+}	0.051	0.054	0.02	0.268	0.178	0.135	0.738	0.704
Mn	0.018	0.005	0.005	0.006	0.002	---	0.003	0.002
Mg	0.039	0.068	0.018	0.75	0.836	0.869	1.246	1.279
Ca	1.887	1.871	1.953	0.974	0.983	0.991	0.009	0.013
Na	---	---	0.002	---	---	0.003	0.001	0.001
K	---	---	---	---	---	---	---	---
Sum	2	2	2	2	2	2	2	2
$Fe^{\#}$	0.57	0.44	0.53	0.26	0.18	0.13	0.37	0.36

Table S1: continued

Mineral	Ep	Ep	Ep	Pa	Pa	Ph	Chl	Chl	Chl
Location	In GrtIII	In Dol	In M	In Grt I	In Dol	In M	In Grt I	In Grt II	In Grt F
SiO_2	39.96	40.29	39.47	47.87	47.6	47.8	27.85	27.01	35.48
TiO_2	0.03	0.13	---	0.03	0.08	0.24	0.06	0.10	0.17
Al_2O_3	32.39	30.49	32.94	40.66	41.05	23.17	18.69	21.73	14.11
Cr_2O_3	0.06	0.08	0.08	0.08	0.04	4.09	0.15	0.13	0.10
FeO	2.48	3.09	0.93	0.58	0.30	5.98	23.97	22.06	20.49
MnO	0.04	0.03	---	0.01	0.01	0.35	0.21	0.31	0.17
MgO	---	0.07	0.05	0.07	0.04	2.37	15.49	15.59	16.26
CaO	23.85	23.35	24.28	0.28	0.80	1.02	0.12	0.30	0.32
Na_2O	---	0.01	---	6.39	5.27	0.67	0.05	0.37	0.17
K_2O	0.02	---	---	0.31	0.73	6.13	0.01	0.06	0.32
Totals	98.83	97.54	97.75	96.28	95.92	91.82	86.60	87.66	87.59
O	12.5	12.5	12.5	11	11	11	14	14	14
Si	3.014	3.074	3.000	3.005	2.995	3.327	2.942	2.792	3.586
Ti	0.002	0.007	---	0.001	0.004	0.013	0.005	0.008	0.013
Al	2.880	2.743	2.952	3.009	3.045	1.902	2.328	2.649	1.681
Cr	0.004	0.005	0.005	0.004	0.002	0.225	0.013	0.011	0.008
Fe^{3+}	0.115	0.195	0.043	0.021	0.011	0.244	---	---	---
Fe^{2+}	0.041	0.002	0.016	0.009	0.005	0.104	2.118	1.907	1.732
Mn	0.003	0.002	---	0.001	0.001	0.021	0.019	0.027	0.015
Mg	---	0.008	0.006	0.007	0.004	0.246	2.439	2.402	2.449
Ca	1.927	1.909	1.978	0.019	0.054	0.076	0.014	0.033	0.035
Na	---	0.001	---	0.778	0.643	0.090	0.010	0.074	0.033
K	0.002	---	---	0.025	0.059	0.544	0.001	0.008	0.041
Sum	7.987	7.947	8	6.878	6.822	6.793	9.888	9.911	9.594
Ps	0.04	0.07	0.01						

Table S1: continued

Mineral	Law	Brs C	Brs M	Brs R	Wnc	Hbl	Ab
Location	In GrtIII	In M	In M	In M	In M	In M	In M
SiO_2	40.38	49.35	49.25	47.47	53.11	49.65	68.47
TiO_2	0.03	0.13	0.17	0.29	0.10	0.18	---
Al_2O_3	30.24	10.18	10.19	11.32	6.74	9.42	19.87
Cr_2O_3	0.26	0.14	0.08	0.11	0.03	0.11	---
FeO	1.02	11.25	11.65	13.53	8.54	13.26	0.09
MnO	---	0.07	0.04	0.07	0.01	0.08	0.03
MgO	0.01	12.5	11.99	10.58	15.53	11.62	---
CaO	17.39	8.51	8.38	8.85	9.27	10.13	0.51
Na_2O	0.13	3.26	3.09	3.32	2.79	2.75	11.64
K_2O	0.07	0.25	0.22	0.17	0.11	0.07	0.03
Totals	89.38	95.64	95.06	95.71	96.23	97.27	100.64
Oxygens	8	23	23	23	23	23	8
Si	2.104	7.179	7.209	7.007	7.561	7.209	2.976
Ti	---	0.014	0.019	0.032	0.011	0.020	---
Al	1.857	1.746	1.758	1.970	1.131	1.613	1.018
Cr	0.011	0.016	0.009	0.013	0.003	0.013	---
Fe^{3+}	---	0.149	0.147	0.100	0.067	---	0.003
Fe^{2+}	0.037	1.220	1.279	1.570	0.950	1.610	---
Mn	---	0.009	0.005	0.009	0.001	0.010	0.001
Mg	0.001	2.710	2.616	2.327	3.295	2.515	---
Ca	0.971	1.326	1.314	1.400	1.414	1.576	0.024
Na	0.013	0.920	0.877	0.950	0.770	0.774	0.981
K	---	0.046	0.041	0.032	0.020	0.013	0.002
Sum	5.000	15.335	15.274	15.410	15.245	15.352	5.005

Note: Mineral abbreviation: Grt, garnet; Omp, omphacite; Cal, calcite; Dol, dolomite; Mgs, magnesite; Ep, epidote; Pa, paragonite; Ph, phengite; Chl, chlorite; Law, lawsonite; Brs, barroisite; Wnc, winchite; Hbl, hornblende; Ab, albite. Location abbreviation: C, core; M, mantle; R, rim; ‘Ar Dol’ means around dolomite; ‘In M’ means in matrix; In Grt F means in garnet fracture. X_{alm} = Fe^{2+}/(Ca+Mg+Fe^{2+}+Mn), X_{grs} = Ca/(Ca+Mg+Fe^{2}+Mn), X_{py} = Mg/(Ca+Mg+Fe^{2+}+Mn), X_{sps} =Mn/(Ca+Mg+Fe^{2+}+Mn). ‘---’ means that the content is below the detection limit.

Table S2. The carbon and hydrogen isotope compositions in the fluid inclusions in eclogites from the Western Tianshan subduction zone

Sample	Host mineral	$\delta^{13}C_{VPDB}$ (‰)	$\delta^{2}H_{VSMOW}$ (‰)
HB142-8	Grt	-30.2	-363.1
	Omp	-29.3	-367.4
HB142-1	Grt	-29.7	-370.6
	Omp	-30.2	-363.7
HB142-2	Grt	-28.6	-379.5
	Omp	-30.0	-359.5
HB142-4	Grt	-30.8	-383.0
	Omp	-30.1	-375.6
HB147-6	Grt	-30.9	-371.1
	Omp	-30.7	-373.5

Table S3. Representative chemical compositions of garnet in eclogite HB142-8, analyzed using Flank Method

Sample	Grt I	Grt II	Grt III	Grt IV
SiO_2	38.65	39.15	38.89	39.38
TiO_2	0.52	0.10	0.07	0.06
Al_2O_3	20.62	21.02	21.13	21.54
Cr_2O_3	0.36	0.05	0.05	0.10
FeO	29.37	31.02	27.09	24.71
MnO	1.16	0.97	0.80	0.43
NiO	0.02	0.02	0.01	0.01
MgO	3.84	4.41	3.18	4.17
CaO	6.39	4.40	9.39	10.53
Na_2O	0.25	0.26	0.23	0.14
Total	101.21	101.43	100.88	101.08
Si	3.02	3.05	3.03	3.03
Ti	0.03	0.01	0.00	0.00
Al	1.90	1.93	1.94	1.95
Cr	0.02	0.00	0.00	0.01
Fe	1.92	2.02	1.77	1.59
Mn	0.08	0.06	0.05	0.03
Ni	0.00	0.00	0.00	0.00
Mg	0.45	0.51	0.37	0.48
Ca	0.54	0.37	0.78	0.87
Na	0.04	0.04	0.04	0.02
Total	8.00	8.00	8.00	7.99
$Fe^{3+}/\sum Fe$	0.025	0.026	0.026	0.029
FeO	28.62	30.20	26.38	23.99
Fe_2O_3	0.83	0.91	0.79	0.79

Table S4. fO_2 calculated for selected P-T conditions

	**T (C)**	**P (kbar)**	**fO_2 (log)**	**FMQ (log)**	**logFMQ**
Grt Ⅰ	520	22.5	-22.80	-20.37	-2.44
Grt Ⅱ	550	33.0	-21.64	-18.12	-3.51
Grt Ⅲ	570	27.5	-20.27	-18.09	-2.18
Grt Ⅳ	580	23.0	-19.27	-18.26	-1.01

Table S5. The effective bulk-rock compositions (wt%) used for calculating in the P-T pseudosection in Figure 3

	**Na_2O**	**MgO**	**Al_2O_3**	**SiO_2**	**K_2O**	**CaO**	**MnO**	**FeO**	**Fe_2O_3**	**H_2O**	**CO_2**
Figure 3a	3.02	5.62	16.30	46.60	0.03	11.52	0.1	6.89	2.33	5.77	2.52
Figure 3b	1.67	8.68	13.39	44.53	0.03	13.45	0.08	6.03	2.03	6.56	3.55

Table S6. Deep Earth Water thermodynamic modelling of carbon species and proportion in aqueous fluids from Western Tianshan cold subduction zone

P-T condition	450°C & 1.5GPa		510°C & 2.5GPa		550 °C & 3.5GPa		570°C & 3.0GPa		580°C & 2.5GPa		550°C & 2.0GPa	
fO_2 condition	QFM-1		QFM-2.4		QFM-3.5		QFM-2.2		QFM-1		QFM+1	
Carbon species	molal conc	mol %	molal conc	mol %	molal conc	mol %	molal conc	mol %	molal conc	mol %	molal conc	mol %
CH_4(AQ)	4.61E+00	61.01	9.38E-01	97.31	1.51E+00	96.42	4.73E-02	15.57	3.40E+00	9.48	0	0.00
ETHANE(AQ)	6.60E-02	0.87	4.94E-03	0.51	3.06E-02	1.95	0	0.00	1.41E-01	0.39	0	0.00
FE(HCOO)+	3.86E-01	5.11	1.70E-02	1.76	1.08E-02	0.69	8.41E-02	27.67	4.72E+00	13.17	4.20E-01	1.34
H_2CO_3(AQ)	5.67E-01	7.51	3.26E-03	0.34	0.00E+00	0.00	7.11E-02	23.40	1.25E+01	34.83	1.63E+01	51.70
CO_2(AQ)	1.03E+00	13.57	0.00E+00	0.00	0.00E+00	0.00	3.41E-02	11.22	7.20E+00	20.07	1.41E+01	44.93
$NAHCO_3$(AQ)	3.85E-01	5.09	0.00E+00	0.00	0.00E+00	0.00	2.76E-02	9.07	1.74E+00	4.85	6.88E-02	0.22
$CA(HCO_3)^+$	2.22E-02	0.29	3.04E-04	0.03	0.00E+00	0.00	7.41E-03	2.44	3.63E-01	1.01	2.85E-01	0.91
$CA(HCOO)^+$	3.60E-03	0.05	4.83E-04	0.05	7.88E-04	0.05	5.36E-03	1.76	2.02E-01	0.56	8.88E-03	0.03
$CH3CH2COO^-$	9.87E-02	1.31	0.00E+00	0.00	1.40E-02	0.89	9.52E-04	0.31	4.65E+00	12.98	0.00E+00	0.00
HCO_3^-	3.13E-01	4.15	0.00E+00	0.00	0.00E+00	0.00	1.36E-02	4.49	5.27E-01	1.47	2.76E-01	0.88
$HCOO^-$	7.85E-02	1.04	0.00E+00	0.00	0.00E+00	0.00	1.24E-02	4.06	4.24E-01	1.18	0.00E+00	0.00

Table S7. The CH_4 flux released from mafic eclogites in subduction zones

**Subduction zone**	**H_2O**	**CH_4**	**[Weight of total eclogites subducted annually]**						**CH_4 flux**	**CH_4 flux**	**C flux**
	**Wt. %**	**mol/kg H_2O**	**length, m**	**depth, m**	**rate, m/y**	**m^3/y**	**Density, kg/m^3**	**kg/y**	**mol/y**	**Mt/y**	**Mt/y**
The modern subduction zones worldwide	4	2.353	4.45E+07	1000	0.054	2.40E+09	3.00E+03	7.20E+12	6.78E+11	1.08E+01	8.14E+00
The ancient Southern Tianshan	4	2.353	2.00E+06	1000	0.054	1.08E+08	3.00E+03	3.24E+11	3.05E+10	4.88E-01	3.66E-01
The cases of global ancient cold and reduced subduction zones	4	2.353	7.20E+06	1000	0.054	3.89E+08	3.00E+03	1.17E+12	1.10E+11	1.76E+00	1.32E+00

Note: the parameters of [weight of total eclogites] are based on Kelemen and Manning, 2015.

Table S8. The carbon and hydrogen isotope compositions of the standard samples

**No.**	**Materials**	**Certified Value (‰)**		**Measured Value (‰)**	
		**$\delta^{13}C_{VPDB}$**	**$\delta^{2}H_{VSMOW}$**	**$\delta^{13}C_{VPDB}$**	**$\delta^{2}H_{VSMOW}$**
IsoRM 201	CH_4	-26.60	-334.3	-26.57	-334.9
IsoRM 202	CH_4	-40.17	-162.3	-40.20	-163.3
IsoRM 203	CH_4	-68.31	-231.3	-68.28	-229.6

Table S9. The input data files used for calculating Fig. 5

Fig.5a

```
log f02 at QFM-1

endit.
     tempc=  .45000E+03
       rho=  .10234E+01    tdspkg=   .00000E+00    tdspl=   .00000E+00
       fep= -.25100E+02    uredox=
     tolbt=  .00000E+00     toldl=   .00000E+00   tolsat=   .00000E+00
    itermx= 99
              1   2   3   4   5   6   7   8   9  10
  iopt1-10=   0   0   0   2   0   0   0   0   0   0
  iopg1-10=   0  -1   0   0   1   0   0   0   0   0
  iopr1-10=   0   2   0   0   0   0   1   0   0   0
 iopr11-20=   0   0   0   0   0   0   0   0   0   0
  iodb1-10=   0   0   0   0   0   0   0   0   0   0
     uebal= H+
    uacion= CL-
     nxmod=  5
   species= DIASPORE
      type=  1            option= -1            xlkmod=  .00000E+00
   species= PYROPHYLLITE
      type=  1            option= -1            xlkmod=  .00000E+00
   species= SEPIOLITE
      type=  1            option= -1            xlkmod=  .00000E+00
   species= CA(OH)+
      type=  0            option= -1            xlkmod=  .00000E+00
   species= MGSIC+
      type=  0            option= -1            xlkmod=  .00000E+00
data file master species= NA+
   switch with species=
   jflag= 19   csp=   .00000E+00
          uphas1= GLAUCOPHANE               uphas2=
data file master species= CL-
   switch with species=
   jflag=  0   csp=   .50000E+00
data file master species= CA++
   switch with species=
   jflag= 20   csp=   .00000E+00
          uphas1= GARNET(SS)           u    uphas2=
data file master species= CO3--
   switch with species=
   jflag= 19   csp=   .00000E+00
          uphas1= DOLOMITE                  uphas2=
data file master species= H+
   switch with species=
   jflag= 16   csp=  -.40000E+01
data file master species= H4SIO4(AQ)
   switch with species=
   jflag= 19   csp=   .00000E+00
          uphas1= QUARTZ-ALPHA              uphas2=
data file master species= MG++
   switch with species=
   jflag= 19   csp=   .00000E+00
          uphas1= CLINOCHLORE               uphas2=
data file master species= FE++
   switch with species=
   jflag= 20   csp=   .00000E+00
          uphas1= GARNET(SS)           u    uphas2=
data file master species= AL+++
   switch with species=
   jflag= 19   csp=   .00000E+00
          uphas1= LAWSONITE                 uphas2=
endit.
 solid solutions
   GARNET(SS)
 component     mole fraction
      PYROPE                          .1000
      ALMANDINE                       .6000
      GROSSULAR                       .3000
      endit.
   endit.
```

Fig.5b

```
510 C & 25 kbar

log f02 at QFM-2.4

endit.
     tempc=  .51000E+03
       rho=  .10234E+01    tdspkg=   .00000E+00    tdspl=   .00000E+00
       fep= -.22800E+02    uredox=
     tolbt=  .00000E+00     toldl=   .00000E+00   tolsat=   .00000E+00
    itermx= 99
              1   2   3   4   5   6   7   8   9  10
  iopt1-10=   0   0   0   2   0   0   0   0   0   0
  iopg1-10=   0  -1   0   0   1   0   0   0   0   0
  iopr1-10=   0   2   0   0   0   0   1   0   0   0
 iopr11-20=   0   0   0   0   0   0   0   0   0   0
  iodb1-10=   0   0   0   0   0   0   0   0   0   0
     uebal= H+
    uacion= CL-
     nxmod=  5
   species= DIASPORE
      type=  1            option= -1            xlkmod=  .00000E+00
   species= PYROPHYLLITE
      type=  1            option= -1            xlkmod=  .00000E+00
   species= SEPIOLITE
      type=  1            option= -1            xlkmod=  .00000E+00
   species= CA(OH)+
      type=  0            option= -1            xlkmod=  .00000E+00
   species= MGSIC+
      type=  0            option= -1            xlkmod=  .00000E+00
data file master species= NA+
   switch with species=
   jflag= 19   csp=   .00000E+00
          uphas1= JADEITE                   uphas2=
data file master species= CL-
   switch with species=
   jflag=  0   csp=   .50000E+00
data file master species= CA++
   switch with species=
   jflag= 20   csp=   .00000E+00
          uphas1= GARNET(SS)           u    uphas2=
data file master species= CO3--
   switch with species=
   jflag= 19   csp=   .00000E+00
          uphas1= GRAPHITE                  uphas2=
data file master species= H+
   switch with species=
   jflag= 16   csp=  -.40000E+01
data file master species= H4SIO4(AQ)
   switch with species=
   jflag= 19   csp=   .00000E+00
          uphas1= QUARTZ-ALPHA              uphas2=
data file master species= MG++
   switch with species=
   jflag= 20   csp=   .00000E+00
          uphas1= GARNET(SS)           u    uphas2=
data file master species= FE++
   switch with species=
   jflag= 20   csp=   .00000E+00
          uphas1= GARNET(SS)           u    uphas2=
data file master species= AL+++
   switch with species=
   jflag= 19   csp=   .00000E+00
          uphas1= LAWSONITE                 uphas2=
endit.
 solid solutions
   GARNET(SS)
 component     mole fraction
      PYROPE                          .1400
      ALMANDINE                       .6100
      GROSSULAR                       .1900
      endit.
   endit.
```

Fig.5c

```
550 C & 35 kbar

log f02 at QFM-3.5

endit.
     tempc=  .55000E+03
       rho=  .10234E+01    tdspkg=   .00000E+00    tdspl=   .00000E+00
       fep= -.21400E+02    uredox=
     tolbt=  .00000E+00     toldl=   .00000E+00   tolsat=   .00000E+00
    itermx= 99
              1   2   3   4   5   6   7   8   9  10
  iopt1-10=   0   0   0   2   0   0   0   0   0   0
  iopg1-10=   0  -1   0   0   1   0   0   0   0   0
  iopr1-10=   0   2   0   0   0   0   1   0   0   0
 iopr11-20=   0   0   0   0   0   0   0   0   0   0
  iodb1-10=   0   0   0   0   0   0   0   0   0   0
     uebal= H+
    uacion= CL-
     nxmod=  5
   species= DIASPORE
      type=  1            option= -1            xlkmod=  .00000E+00
   species= PYROPHYLLITE
      type=  1            option= -1            xlkmod=  .00000E+00
   species= SEPIOLITE
      type=  1            option= -1            xlkmod=  .00000E+00
   species= CA(OH)+
      type=  0            option= -1            xlkmod=  .00000E+00
   species= MGSIC+
      type=  0            option= -1            xlkmod=  .00000E+00
data file master species= NA+
   switch with species=
   jflag= 19   csp=   .00000E+00
          uphas1= JADEITE                   uphas2=
data file master species= CL-
   switch with species=
   jflag=  0   csp=   .50000E+00
data file master species= CA++
   switch with species=
   jflag= 20   csp=   .00000E+00
          uphas1= GARNET(SS)           u    uphas2=
data file master species= CO3--
   switch with species=
   jflag= 19   csp=   .00000E+00
          uphas1= GRAPHITE                  uphas2=
data file master species= H+
   switch with species=
   jflag= 16   csp=  -.40000E+01
data file master species= H4SIO4(AQ)
   switch with species=
   jflag= 19   csp=   .00000E+00
          uphas1= COESITE                   uphas2=
data file master species= MG++
   switch with species=
   jflag= 20   csp=   .00000E+00
          uphas1= GARNET(SS)           u    uphas2=
data file master species= FE++
   switch with species=
   jflag= 20   csp=   .00000E+00
          uphas1= GARNET(SS)           u    uphas2=
data file master species= AL+++
   switch with species=
   jflag= 19   csp=   .00000E+00
          uphas1= LAWSONITE                 uphas2=
endit.
 solid solutions
   GARNET(SS)
 component     mole fraction
      PYROPE                          .1400
      ALMANDINE                       .7000
      GROSSULAR                       .1300
      endit.
   endit.
```

Fig.5d

```
570 C & 30 kbar

log f02 at QFM-2.2

endit.
     tempc=  .55000E+03
       rho=  .10234E+01    tdspkg=   .00000E+00    tdspl=   .00000E+00
       fep= -.19820E+02    uredox=
     tolbt=  .00000E+00     toldl=   .00000E+00   tolsat=   .00000E+00
    itermx= 99
              1   2   3   4   5   6   7   8   9  10
  iopt1-10=   0   0   0   2   0   0   0   0   0   0
  iopg1-10=   0  -1   0   0   1   0   0   0   0   0
  iopr1-10=   0   2   0   0   0   0   1   0   0   0
 iopr11-20=   0   0   0   0   0   0   0   0   0   0
  iodb1-10=   0   0   0   0   0   0   0   0   0   0
     uebal= H+
    uacion= CL-
     nxmod=  5
   species= DIASPORE
      type=  1            option= -1            xlkmod=  .00000E+00
   species= PYROPHYLLITE
      type=  1            option= -1            xlkmod=  .00000E+00
   species= SEPIOLITE
      type=  1            option= -1            xlkmod=  .00000E+00
   species= CA(OH)+
      type=  0            option= -1            xlkmod=  .00000E+00
   species= MGSIC+
      type=  0            option= -1            xlkmod=  .00000E+00
data file master species= NA+
   switch with species=
   jflag= 19   csp=   .00000E+00
          uphas1= JADEITE                   uphas2=
data file master species= CL-
   switch with species=
   jflag=  0   csp=   .50000E+00
data file master species= CA++
   switch with species=
   jflag= 20   csp=   .00000E+00
          uphas1= GARNET(SS)           u    uphas2=
data file master species= CO3--
   switch with species=
   jflag= 19   csp=   .00000E+00
          uphas1= GRAPHITE                  uphas2=
data file master species= H+
   switch with species=
   jflag= 16   csp=  -.40000E+01
data file master species= H4SIO4(AQ)
   switch with species=
   jflag= 19   csp=   .00000E+00
          uphas1= COESITE                   uphas2=
data file master species= MG++
   switch with species=
   jflag= 20   csp=   .00000E+00
          uphas1= GARNET(SS)           u    uphas2=
data file master species= FE++
   switch with species=
   jflag= 20   csp=   .00000E+00
          uphas1= GARNET(SS)           u    uphas2=
data file master species= AL+++
   switch with species=
   jflag= 19   csp=   .00000E+00
          uphas1= LAWSONITE                 uphas2=
endit.
 solid solutions
   GARNET(SS)
 component     mole fraction
      PYROPE                          .1400
      ALMANDINE                       .6200
      GROSSULAR                       .2200
      endit.
   endit.
```

Fig.5e

```
580 C & 25 kbar

log f02 at QFM-1

endit.
     tempc=  .58000E+03
       rho=  .10234E+01    tdspkg=   .00000E+00    tdspl=   .00000E+00
       fep= -.19100E+02    uredox=
     tolbt=  .00000E+00     toldl=   .00000E+00   tolsat=   .00000E+00
    itermx= 99
              1   2   3   4   5   6   7   8   9  10
  iopt1-10=   0   0   0   2   0   0   0   0   0   0
  iopg1-10=   0  -1   0   0   1   0   0   0   0   0
  iopr1-10=   0   2   0   0   0   0   1   0   0   0
 iopr11-20=   0   0   0   0   0   0   0   0   0   0
  iodb1-10=   0   0   0   0   0   0   0   0   0   0
     uebal= H+
    uacion= CL-
     nxmod=  5
   species= DIASPORE
      type=  1            option= -1            xlkmod=  .00000E+00
   species= PYROPHYLLITE
      type=  1            option= -1            xlkmod=  .00000E+00
   species= SEPIOLITE
      type=  1            option= -1            xlkmod=  .00000E+00
   species= CA(OH)+
      type=  0            option= -1            xlkmod=  .00000E+00
   species= MGSIC+
      type=  0            option= -1            xlkmod=  .00000E+00
data file master species= NA+
   switch with species=
   jflag= 19   csp=   .00000E+00
          uphas1= JADEITE                   uphas2=
data file master species= CL-
   switch with species=
   jflag=  0   csp=   .50000E+00
data file master species= CA++
   switch with species=
   jflag= 20   csp=   .00000E+00
          uphas1= GARNET(SS)           u    uphas2=
data file master species= CO3--
   switch with species= CH4(AQ)
   jflag= 19   csp=   .00000E+00
          uphas1= MAGNESITE                 uphas2=
data file master species= H+
   switch with species=
   jflag= 16   csp=  -.40000E+01
data file master species= H4SIO4(AQ)
   switch with species=
   jflag= 19   csp=   .00000E+00
          uphas1= QUARTZ-ALPHA              uphas2=
data file master species= MG++
   switch with species=
   jflag= 20   csp=   .00000E+00
          uphas1= GARNET(SS)           u    uphas2=
data file master species= FE++
   switch with species=
   jflag= 20   csp=   .00000E+00
          uphas1= GARNET(SS)           u    uphas2=
data file master species= AL+++
   switch with species=
   jflag= 19   csp=   .00000E+00
          uphas1= LAWSONITE                 uphas2=
endit.
 solid solutions
   GARNET(SS)
 component     mole fraction
      PYROPE                          .1500
      ALMANDINE                       .5300
      GROSSULAR                       .3000
      endit.
   endit.
```

Fig.5f

```
550 C & 20 kbar

log f02 at QFM-1

endit.
     tempc=  .55000E+03
       rho=  .10234E+01    tdspkg=   .00000E+00    tdspl=   .00000E+00
       fep= -.18400E+02    uredox=
     tolbt=  .00000E+00     toldl=   .00000E+00   tolsat=   .00000E+00
    itermx= 99
              1   2   3   4   5   6   7   8   9  10
  iopt1-10=   0   0   0   2   0   0   0   0   0   0
  iopg1-10=   0  -1   0   0   1   0   0   0   0   0
  iopr1-10=   0   2   0   0   0   0   1   0   0   0
 iopr11-20=   0   0   0   0   0   0   0   0   0   0
  iodb1-10=   0   0   0   0   0   0   0   0   0   0
     uebal= H+
    uacion= CL-
     nxmod=  5
   species= DIASPORE
      type=  1            option= -1            xlkmod=  .00000E+00
   species= PYROPHYLLITE
      type=  1            option= -1            xlkmod=  .00000E+00
   species= SEPIOLITE
      type=  1            option= -1            xlkmod=  .00000E+00
   species= CA(OH)+
      type=  0            option= -1            xlkmod=  .00000E+00
   species= MGSIC+
      type=  0            option= -1            xlkmod=  .00000E+00
data file master species= NA+
   switch with species=
   jflag= 19   csp=   .00000E+00
          uphas1= PARAGONITE                uphas2=
data file master species= CL-
   switch with species=
   jflag=  0   csp=   .50000E+00
data file master species= CA++
   switch with species=
   jflag= 20   csp=   .00000E+00
          uphas1= GARNET(SS)           u    uphas2=
data file master species= CO3--
   switch with species=
   jflag= 19   csp=   .00000E+00
          uphas1= MAGNESITE                 uphas2=
data file master species= H+
   switch with species=
   jflag= 16   csp=  -.40000E+01
data file master species= H4SIO4(AQ)
   switch with species=
   jflag= 19   csp=   .00000E+00
          uphas1= QUARTZ-ALPHA              uphas2=
data file master species= MG++
   switch with species=
   jflag= 20   csp=   .00000E+00
          uphas1= GARNET(SS)           u    uphas2=
data file master species= FE++
   switch with species=
   jflag= 20   csp=   .00000E+00
          uphas1= GARNET(SS)           u    uphas2=
data file master species= AL+++
   switch with species=
   jflag= 19   csp=   .00000E+00
          uphas1= CLINOZOISITE              uphas2=
endit.
 solid solutions
   GARNET(SS)
 component     mole fraction
      PYROPE                          .1800
      ALMANDINE                       .5000
      GROSSULAR                       .3000
      endit.
   endit.
```

Supplementary References

Bebout GE, Fogel ML and Cartigny P. Nitrogen: Highly volatile yet surprisingly compatible. *Elements* 2013; **9:** 333–338.

Vitale Brovarone A, Martinez I and Elmaleh A *et al.* Massive production of abiotic methane during subduction evidenced in metamorphosed ophicarbonates from the Italian Alps. *Nat Commun* 2017; **8**: 14134.

Vitale Brovarone A, Sverjensky DA and Piccoli F *et al.* Subduction hides high-pressure sources of energy that may feed the deep subsurface biosphere. *Nat Commun* 2020; **11**: 3880.

Boutier A, Vitale Brovarone A and Martinez I *et al*. High pressure serpentinization and abiotic methane formation in metaperidotite from the Appalachian subduction, northern Vermont. *Lithos* 2021; **396:** 106190.

Coggon R and Holland TJB. Mixing properties of phengitic micas and revised garnet–phengite thermobarometers. *J Metamorph Geol* 2002; **20:** 683–696.

Connolly JAD and Trommsdorff V. Petrogenetic grids for metacarbonate rocks: pressure-temperature phase-diagram projection for mixed-volatile systems. *Contrib to Mineral Petrol* 1991; **108**: 93–105.

Dale J, Powell R and White RW *et al.* Athermodynamic model for Ca-Na clinoamphiboles in Na2O-CaO-FeO-MgO-Al2O3-SiO2-H2O-O for petrological calculations. *J Metamorph Geol* 2005; **23**: 771–791.

Diener JFA and Powell R. Revised activity–composition models for clinopyroxene and amphibole. *J Metamorph Geol* 2012; **30**: 131–142.

Franzolin E, Schmidt MW and Poli S. Ternary Ca-Fe-Mg carbonates: subsolidus phase relations at 3.5 GPa and a thermodynamic solid solution model including order/disorder. *Contrib to Mineral Petrol* 2011; **161**: 213–227.

Holland TJB and Powell R. An internally consistent thermodynamic data set for phases of petrological interest. *J Metamorph Geol* 1998; **16**: 309–343.

Kelemen PB and Manning CE. Reevaluating carbon fluxes in subduction zones, what goes down, mostly comes up. *Proc Natl Acad Sci USA* 2015; **112**: 3997–4006.

Peng WG, Zhang LF and Tumiati S *et al.* Abiotic methane generation through reduction of serpentinite-hosted dolomite: implications for carbon mobility in subduction zones. *Geochim Cosmochim Acta*

2021; **311**: 119–140.

Schmidt MW and Poli S. Devolatilization during subduction. In: Holland HD (ed.). *Treatise on Geochemistry*, Elsevier Science Ltd, 2014; 4.19, 669–701.

Song SG, Su L and Niu YL *et al*. CH_4 inclusions in orogenic harzburgite: Evidence for reduced slab fluids and implication for redox melting in mantle wedge. *Geochim Cosmochim Acta* 2009; **73**: 1737–1754.

Tao RB, Zhang LF and Tian M *et al.* Formation of abiotic hydrocarbon from reduction of carbonate in subduction zones: Constraints from petrological observation and experimental simulation. *Geochim Cosmochim Acta* 2018; **239**: 390–408.

White RW, Powell R and Holland TJB. Progress relating to calculation of partial melting equilibria formetapelites and felsic gneisses. *J Metamorph Geol* 2007; **25**: 511–527.

Zhang LF, Wang Y and Zhang LJ *et al*. Ultrahigh Pressure Metamorphism and Tectonic Evolution of Southwestern Tianshan Orogenic Belt, China: A Comprehensive Review. *Geol Soc Spec Publ* 2018; **474**: 133–152.
